# Population-based study of the durability of humoral immunity after SARS-CoV-2 infection

**DOI:** 10.3389/fimmu.2023.1242536

**Published:** 2023-10-05

**Authors:** David Peterhoff, Simon Wiegrebe, Sebastian Einhauser, Arisha J. Patt, Stephanie Beileke, Felix Günther, Philipp Steininger, Hans H. Niller, Ralph Burkhardt, Helmut Küchenhoff, Olaf Gefeller, Klaus Überla, Iris M. Heid, Ralf Wagner

**Affiliations:** ^1^Institute of Medical Microbiology and Hygiene, Molecular Microbiology (Virology), University of Regensburg, Regensburg, Germany; ^2^Institute of Clinical Microbiology and Hygiene, University Hospital Regensburg, Regensburg, Germany; ^3^Department of Genetic Epidemiology, University of Regensburg, Regensburg, Germany; ^4^Statistical Consulting Unit StaBLab, Department of Statistics, Ludwig-Maximilians-Universität (LMU) Munich, Munich, Germany; ^5^Institute of Clinical and Molecular Virology, University Hospital Erlangen, Friedrich-Alexander-Universität Erlangen-Nürnberg, Erlangen, Germany; ^6^Institute of Clinical Chemistry and Laboratory Medicine, University Hospital Regensburg, Regensburg, Germany; ^7^Department of Medical Informatics, Biometry and Epidemiology, Friedrich-Alexander-Universität Erlangen-Nürnberg (FAU), Erlangen, Germany

**Keywords:** SARS-CoV-2, COVID-19, longitudinal, durability, population-based, antibody, neutralization, avidity

## Abstract

SARS-CoV-2 antibody quantity and quality are key markers of humoral immunity. However, there is substantial uncertainty about their durability. We investigated levels and temporal change of SARS-CoV-2 antibody quantity and quality. We analyzed sera (8 binding, 4 avidity assays for spike-(S-)protein and nucleocapsid-(N-)protein; neutralization) from 211 seropositive unvaccinated participants, from the population-based longitudinal TiKoCo study, at three time points within one year after infection with the ancestral SARS-CoV-2 virus. We found a significant decline of neutralization titers and binding antibody levels in most assays (linear mixed regression model, p<0.01). S-specific serum avidity increased markedly over time, in contrast to N-specific. Binding antibody levels were higher in older versus younger participants – a difference that disappeared for the asymptomatic-infected. We found stronger antibody decline in men versus women and lower binding and avidity levels in current versus never-smokers. Our comprehensive longitudinal analyses across 13 antibody assays suggest decreased neutralization-based protection and prolonged affinity maturation within one year after infection.

## 1 Introduction

Infection-induced antibodies are key markers of the humoral immunity. Their quantity and quality influence their capacity to protect against re-infection (1). Systemic viral infections elicit protective antibodies that are present for years to decades, whereas the persistence of protective antibodies after mucosal viral infections is often limited to a few months to years (2). Consistent with this, humoral immunity to human seasonal coronavirus infections as well as to the epidemic severe acute respiratory syndrome coronavirus 1 (SARS-CoV-1) and endemic Middle East respiratory syndrome-related coronavirus (MERS-CoV) has been shown to significantly decrease within one year (3–6). In line with this, several studies have recently shown that the humoral immune responses to the currently pandemic severe acute respiratory syndrome coronavirus 2 (SARS-CoV-2) infection wane rapidly. However, although a number of population-based SARS-CoV-2 serosurveillance studies have recently been published (7–15), information regarding the time to and extent of immunity waning after SARS-CoV-2 infection in the general population is still sparse (for a selection of studies see (16–20)). Most published studies so far did not recruit a random sample from a general population, but from blood donors, medical records, or infection registries (21–38) and the generalizability of their findings is thus limited. For a systematic analysis of the temporal course of the humoral immune status after COVID-19, the inclusion of individuals from a wide range of age and disease severities including asymptomatic cases is required. Furthermore, an understanding of the temporal course and waning of immunity of individuals with asymptomatic infections remains highly relevant for societal risk assessment, since these individuals contribute to the spread of the pandemic (39). This can be investigated in population-based studies by measuring occurred infections based on serum-status rather than health-authority reporting.

In particular, the role of sociodemographic factors in the development of antibody levels and their quality over time is a current matter of debate and has not been conclusively investigated by means of representative studies (40). Whether old age is associated with initially higher titers and a subsequent steeper decline, implying a faster waning of immune response, is currently unknown and longitudinal population-based data for this is lacking. There is currently no population-based data as to differences in antibody decline by sex.

Due to the constant evolution of novel SARS-CoV-2 strains with different antigenic properties, a diversification of the induced immune response has already emerged. In addition, widespread vaccination campaigns resulted in the appearance of vaccine-induced antibodies. Consequently, it is increasingly difficult, if not impossible, to recruit vaccination-naïve population-based cohorts of individuals infected with a certain SARS-CoV-2 strain over time in order to study mechanisms of humoral immunity. Thus, only cohorts from the early days of the pandemic can serve as a reference for answering fundamental questions on the characteristics in SARS-CoV-2 host-pathogen interactions.

Here, we analyzed serum samples from the population-based longitudinal Tirschenreuth Kohorte COVID-19 (TiKoCo) study, a representative cohort study of individuals aged at least 14 years from the German Tirschenreuth county (10, 41–43). Three hundred fifty-two of the 4185 study participants became infected with SARS-CoV-2 during a narrow period of 4 weeks from late February to late March 2020. Of these, a nested group of 211 attended the study visits at 3, 8, and 13 months post infection without having received vaccination in the interim (vaccination roll-out end of December 2020 with limited access until April 2021). Of note, within this timeframe of infections until June 2020, no variants of concern (VOC) were present in the study population. To assess the course of SARS-CoV-2 directed humoral immunity in a comprehensive way, we aimed to investigate a full range of antibody measurements. We thus applied 12 assays capturing the quantity and quality of immune response against the two major antigenic determinants – the spike (S) and the nucleocapsid (N) protein – in total as well as mapped down to the level of the important protein domains. To close the gap between immune response and protective effector function, we also applied a neutralization test.

## 2 Materials and methods

### 2.1 Study design

The TiKoCo study is a longitudinal population-based cohort study from the Tirschenreuth county, Bavaria, Germany, as described previously (10, 43). Briefly, a total of 6540 Tirschenreuth county inhabitants were invited, 4203 of whom participated in the baseline (BL) survey in June 2020 (within a period of 19 days). Of these, 3546 and 3391 participated in the follow-up surveys in November 2020 and April 2021, respectively (FU1, FU2; each of the visits extended over 12 days). All participants filled out a standardized questionnaire at each of the three surveys. Blood was drawn for all participants at all three time points.

Here, we analyzed the individuals who participated and had valid antibody measurements at all three time points. Of these, we selected the participants who were antibody-positive according to the latent class analysis (LCA) combining results from three different serological assays at BL (10) and were not vaccinated against COVID-19 (10, 42, 43). The infection wave in spring 2020 was particularly severe in this county, beginning in late February with a peak in mid-March, followed by a strict lockdown that resulted in the number of infections dropping to almost zero thereafter (10, 44). This enabled the analysis of three sera over time up to 13 months after natural infection (10 months after the documented positive antibody status in June 2020) in unvaccinated individuals.

The TiKoCo study was approved by the Ethics Committee of the University of Regensburg (vote 20-1867-101) and the Ethics Committee of the University of Erlangen (vote 248_20 Bc). The study complies with the 1964 Declaration of Helsinki and its later amendments. All participants provided written informed consent.

### 2.2 Questionnaire

The questionnaire was administered at all three surveys and described previously (10). For each of the respective three observation periods, participants were asked about their smoking status (current, ex-, or never smoking), their vaccination status (at FU2, no vaccination available at the time of BL or FU1), previous registered SARS-CoV-2 infection (validated by health authorities) and any COVID-19 related symptoms at the time of the registered infection, and potentially COVID-19 related symptoms during the observation period. Queried symptoms considered potentially COVID-19 related were “breathing problems”, “cough”, “dyspnea”, “fever”, “chills”, “limb pains”, “dysosmia”, “dysgeusia”, “headache”, “exhaustion”, “cold”, or COVID-19 related hospitalization (at least one overnight stay). In our analyses of individuals that were sero-positive at BL, we derived a variable that indicated whether the underlying infection was “symptomatic”, “maybe symptomatic” or “asymptomatic” during the time period when the infection leading to the sero-positivity must have occurred as follows: (i) individuals reported to have been registered as infected (i.e. positive PCR test) until BL and to have had symptoms at that time (“symptomatic”), (ii) individuals had not been registered as infected (thus no infection-date specific report of symptoms available), but the individual had reported any potentially COVID-19 related symptoms until BL (“may be symptomatic”), (iii) individuals had not reported any COVID-19 related symptoms during the time period, and specifically, if they had a PCR-test, they had not reported symptoms at that time (“asymptomatic”).

### 2.3 Serum processing

A total volume of 7.5 ml blood was drawn on every visit by qualified study personnel using a serum monovette (Sarstedt AG Co.KG, Nümbrecht, Germany). Blood draws were organized in three study centers in the Tirschenreuth county, where study participants were invited to participate by blood donation. Serum samples were generated by centrifugation (2000 g, 5 min) at the day of blood donation and stored at -20°C until measurement. Repeated freeze-thaw-cycles of the samples during the measurements were avoided via aliquoting.

### 2.4 Antigen preparation

The design, expression and purification strategy of SARS-CoV-2 spike protein’s receptor-binding domain and its stabilized ectodomain (both based on the sequence of the Wuhan strain) was performed as described earlier (45).

The N-terminal domain (NTD, amino acid 44-174) and the C-terminal domain (CTD, amino acid 258-361) were codon optimized for *Escherichia coli* and synthesized by gene synthesis (GeneArt, Thermo Fisher Scientific). The genes were cloned into the inducible expression-vector pET24a, which adds a C-terminal hexahistidine tag, and were verified by Sanger sequencing. Expression was performed in *E. coli* BL21(DE3). After induction with isopropyl-β-D-thiogalactopyranosid at an optical density at 600 nm (OD_600_) and 6 h expression at 37°C, the cells were disrupted by sonification and a crude extract was generated by pelleting the cell debris by centrifugation (30 min at 4°C and a relative centrifugal force of 20.000). The crude extract was filtered (0.2 µm Filtropur S syringe filters, Sarstedt) loaded on a Ni^2+^-nitrilotriacetic acid column (HisTrap excel, Cytiva) and extensively washed with 10 mM imidazole in phosphate buffered saline (PBS). Elution via a linear imidazole gradient (10 mM to 500 mM imidazole) was performed on an ÄKTA Start chromatography system (Cytiva). Fractions containing the target protein were identified on a reducing sodium dodecyl sulfate polyacrylamide gel electrophoresis as described earlier (45). The target protein containing fractions were pooled, concentrated (Amicon Ultra-15 Centrifugal Filter Units, Millipore Sigma) and buffer exchanged to PBS by Sephadex G-25 resin based gel filtration chromatography (PD-10 column, Cytiva).

### 2.5 Serological tests

The two commercial tests used in this study (S Elecsys and N Elecsys) measure spike- and nucleoprotein-directed total immunoglobulins (Ig) in an electrogenerated chemiluminescence assay (ECLIA) that determines bridging antibodies between two differently modified detector antigens. The used in-house tests are enzyme-linked immunosorbent assays (ELISA), where the respective antigen is directly bound to the surface of the well of a microplate. The ELISA and its modification with different antigens has been characterized in several studies (45–47). Antigens used were the SARS-CoV-2 spike (S) protein’s ectodomain (S ecto) and its receptor-binding domain (S RBD) as well as the SARS-CoV-2 nucleocapsid protein’s (N) N-terminal (N NTD) and C-terminal (N CTD) domain.

The Elecsys Anti-SARS-CoV-2 N and S tests (Roche Diagnostics GmbH, Penzberg, Germany) were operated on the Cobas pro e 801 module according to the manufacturers recommendations, respectively. For quantitative measurements, the samples were diluted 1:10 in PBS if necessary, to stay within the dynamic range of the assay. The in-house ELISA detecting IgG, IgA or IgM antibody responses to the receptor binding domain (RBD) or the ectodomain of the SARS-CoV-2 spike protein was performed as described earlier (45). For the three isotype-specific RBD-ELISAs we determined test-specific cutoff-values and thus provide signal-to-cutoff ratios (S/CO). For the N-ELISAs, purified NTD and CTD were coated at a concentration of 1 µg/ml in PBS to microtiterplates (NUNC Maxisorp 96 well, Thermo Fisher Scientific). Further handling and readout was performed similar to the spike-protein ELISA.

The ELISA for determining the avidity index (AI) of the samples was carried out as described before (48). Briefly, sera were diluted 1:100 in two side by side replicates in 1% fat free milk powder in PBS, containing 0.1% Tween 20 (PBS-T). After the serum binding step, the wells were washed ten times with 200 µl PBS-T. Next, the replicates were treated either with 100 µl of 1.5 M sodium thiocyanate (NaSCN) in PBS or with 100 µl PBS for 15 min at ambient temperature. After a further PBS-T washing step, the ELISA was continued as described. The AI was determined as the ratio of the background corrected absorption (optical density [OD], at 450 nm and 620 nm) of the NaSCN-treated and the PBS-treated sample (see formula below).


$$AI=\frac{OD_{450}-OD_{630}\;\left(\text{NaSCN}\right)}{OD_{450}-OD_{630}\;\left(\text{PBS}\right)}$$

### 2.6 Pseudotype neutralization assay

Neutralization capacity of the sera against SARS-CoV-2 (Wuhan) was evaluated using Vesicular Stomatitis Virus pseudotyped with SARS-CoV-2 spike protein lacking the endoplasmic reticulum retention signal as described before (41, 49). Briefly, pseudoviral titers were determined by limited dilution and fluorescence microscopy.

For neutralization testing, 25,000 fluorescent focus-forming units were incubated for 1 h with 2-fold serum dilutions starting at 1/20 dilutions in triplicates. HEK293T-ACE2^+^ cells were incubated with the mixtures for 20 h, and infected cells were visualized by their luciferase activity using BrightGlo (Promega Corp, Madison, WI, USA). Fifty percent inhibitory concentration values (IC50) were calculated using the GraphPad Prism 9 software (GraphPad Software, San Diego, CA, USA). In case of no measureable neutralization and thus impossible curve fitting, IC50 values were assigned to 0.

### 2.7 Statistical analysis

In a first step, Wilcoxon signed-rank tests were used to test (percentage) changes in titer values at FU1 and FU2, respectively, against BL. By this, we obtained the information whether titer values significantly changed from BL to FU1 or FU2, yet without taking into account age, sex, or smoking status of the participant. Older individuals were potentially expected to have lower antibody response and more rapid titer decline. It was also plausible that sex or smoking status would have an effect on titer levels or titer change.

In order to incorporate age, sex and smoking into this analysis, we employed one Linear Mixed Model (LMM) for each of the 13 assays. Titer values were transformed with the natural logarithm to ensure an approximate normal distribution; for pseudovirus neutralization (ID50), +1 was added to each observation in order to allow for a ln-transformation even of those observations with levels of exactly 0. The outcome values of the LMM were titer measurements over time for all individuals, Y_it_, where i=1,…,n indicates the individuals and t=1,2,3 indicates that the measurement was from BL, FU1, or FU2, respectively. The model included two time-variables as covariates (timeFU1 = 1 if the measurement was from FU1, 0 otherwise; analogously for timeFU2). The covariates age (in years, centered at 50 years), sex (1=women, 0=men), and smoking status (2 variables: smoker_current=1 or 0, smoker_ex=1 or 0, yielding never smokers as reference), as well as their interaction with the time variables were also included into the LMM.

We thus applied 13 LMMs, each for one of the 13 titer levels as outcome:


$$\begin{array}{l} Y_{it}={\text{β}}_{0}+{\text{β}}_{1}*timeFU1_{it}+{\text{β}}_{2}*timeFU2_{it}+{\text{β}}_{3}*age_{i0}+\;{\text{β}}_{4}*sex_{i}\\ \;+{\text{β}}_{5}*smoker\_current_{it}+{\text{β}}_{6}*smoker\_ex_{it}+{\text{β}}_{7}*timeFU1_{it}*age_{i0}\\ \;+{\text{β}}_{8}*timeFU2_{it}*age_{i0}+{\text{β}}_{9}*timeFU1_{it}*sex_{i}+{\text{β}}_{10}*timeFU2_{it}*sex_{i}\\ \;+{\text{β}}_{11}*timeFU1_{it}*smoker\_current_{it}+\;{\text{β}}_{12}*timeFU2_{it}*smoker\_current_{it}\\ \;+{\text{β}}_{13}*timeFU1_{it}*smoker\_ex_{it}+\;{\text{β}}_{14}*timeFU2_{it}*smoker\_ex_{it}+\gamma_{i}+\epsilon_{it}, \end{array}$$

where $\gamma_{i}$ denotes the subject-level random intercept ( $\gamma_{i}∽N(0,\;\sigma_{\gamma }^{2}$ ) and $\epsilon_{it}$ the random error term ( ${\text{ε}}_{it}∽N(0,\sigma^{2}),{\text{ε}}_{it}⫫\gamma_{i}$ )

A significant association of timeFU1 or timeFU2 with $Y_{it}$ can be interpreted as a significant change in titer values between FU1 and BL or between FU2 and BL, respectively, and exp(β) quantifies the change as multiplicative factor. The association of age, sex or smoking status on. represents the effect of these covariates on titer levels; the interaction of age, sex, or smoking status with timeFU1 or timeFU2 can be interpreted as the effect of these covariates on titer change between BL and FU1 or FU2, respectively. In the remainder of this work, we use the term “coefficient” to refer to the model estimates of the β parameters.

We also explored a potential impact of symptomatic versus asymptomatic infections on titer levels as well as their interaction with age. To do so, we classified individuals based on the baseline questionnaire into either “symptomatic” (if PCR-based registered infection and symptoms reported for the time of infection), “maybe symptomatic” (symptoms were reported during the respective time period without ascertainment of the time of infection by PCR-test and thus no specific link of symptoms to the infection) and “asymptomatic” (everyone else, i.e. no symptoms were reported during the respective time period). We then extended the model by adding the following terms:


, $$\beta_{15}*symptomatic_{yes_{i0}}+\beta_{16}*symptomatic_{maybe_{i0}}+\beta_{17}*age_{it}*symptomatic_{yes_{i0}}+\beta_{18}*age_{it}*symptomatic\_maybe_{i0,}$$

where $symptomatic\_yes_{i0}\;$ and $symptomatic\_maybe_{i0}$ are indicator variables taking on the value 1 if individual *i* was categorized as “symptomatic” or “maybe symptomatic” at BL, respectively, and 0 otherwise (making the “asymptomatic” to the reference).

When testing the percentage change from BL to FU1 or FU2 for each of the 13 assays with the Wilcoxon signed rank test or in the LMM, there are multiple statistical tests involved. Since the 13 different assay measurements were partly highly correlated with each other, we derived the number of independent dimensions across these 13 variables by principal component analysis, yielding 5 independent dimensions according to both the elbow method (visual inspection of the number of relevant components using a scree plot (50);) and Kaiser criterion (retaining only those components with eigenvalue ≥ 1 (51)). To judge the Wilcoxon signed rank test as significantly rejecting the null hypothesis for no change or to judge a coefficient in the LMM to be significantly different from zero for any of the 13 titer levels, we applied a Bonferroni-corrected significance level of 0.05 divided by the number of independent dimensions of the 13 assay measurements (i.e. 1% significance level). For the LMM, this does not account for the multiple testing of each of the covariates per multivariable regression model; however, the general recommendation there is to judge the strength of the association by graphical presentation of predicted outcome levels given the coefficients from the model together with corresponding confidence intervals. We present these LMM results accordingly with 99%-confidence intervals.

All statistical analyses were conducted using R software.

## 3 Results

**Figure 1:**
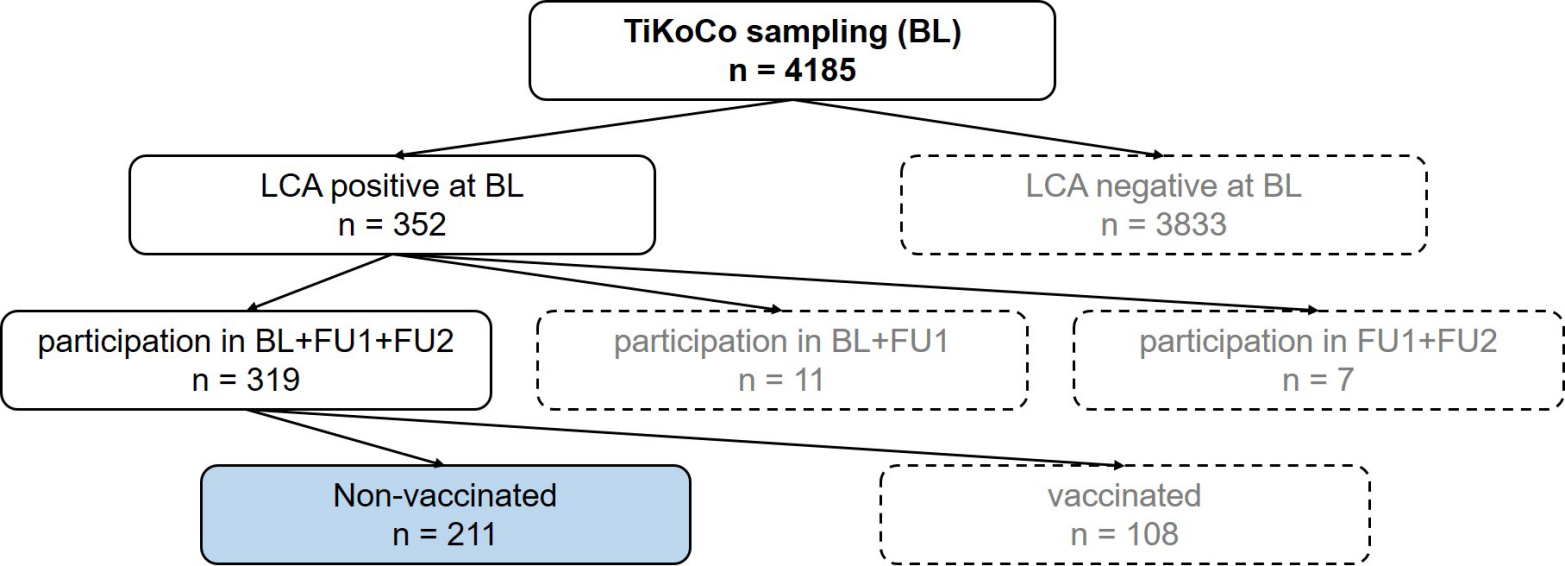
Flow chart to illustrate the selection of the samples. At baseline, 4185 serum samples were collected. Of these samples, 352 were positive in a latent class analysis (LCA) of three SARS-CoV-2 antibody tests. Of these participants, 319 attended the next two visits, follow up 1 (FU1) and follow up 2 (FU2), with 211 participants not vaccinated at the time of FU2. These 211 participants (blue highlighted box) represent the study collective.

We analyzed serum samples from the population-based Tirschenreuth study (TiKoCo) participants, which were collected at three time points from each individual followed longitudinally (BL, June 2020; FU1, November 2020; FU2, April 2021). Here, we included participants who were SARS-CoV-2 seropositive at BL and not vaccinated until FU2 (**Figure 1**). This yielded a total of 211 individuals with serum measurements at three time points longitudinally, with the second and third generated at 5 months and 10 months after the initial sampling at BL. This enabled the investigation of serum measurements at approximately 8 and 13 months after the natural infection had taken place (primarily at the end of February and in March 2020). The analyzed participants were 14 to 92 years old at BL, with 53.6% being women (**Table 1**).

**Table 1:** Participant characteristics at baseline.

	BL (all)	BL (women)	BL (men)
	[n=211]	[n=113]	[n=98]
Mean age (SD)	46.1 (17.3)	45.3 (18.1)	47.2 (16.2)
Median age (IQR)	49.0 (30.5-58.0)	49.0 (29.0-57.0)	49.5 (37.3-58.0)
Min, max age	14.0, 92.0	14.0, 92.0	14.0, 89.0
Age 14-19: % (#)	9.0 (19)	8.0 (9)	10.2 (10)
Age 20-49: % (#)	42.2 (89)	44.2 (50)	39.8 (39)
Age 50-69: % (#)	42.2 (89)	39.8 (45)	44.9 (44)
Age 70+: % (#)	6.6 (14)	8.0 (9)	5.1 (5)
Women: % (#)	53.6 (113)	–	–
Never smoker: % (#)	67.3 (142)	69.9 (79)	64.3 (63)
Ex-smoker: % (#)	23.7 (50)	22.1 (25)	25.5 (25)
Current smoker: % (#)	9.0 (19)	8.0 (9)	10.2 (10)
Symptomatic yes: % (#)	20.9 (44)	20.4 (23)	21.4 (21)
Symptomatic maybe: % (#)	64.9 (137)	65.5 (74)	64.3 (63)
Symptomatic no: % (#)	14.2 (30)	14.2 (16)	14.3 (14)

For the participants at baseline (BL), descriptive statistics are shown. For quantitative variables, mean, standard deviation (SD), median and interquartile range (IQR) are stated; for binary or categorical variables, proportions and absolute numbers are reported. We denote by n the number of individuals in the analyzed sample. Age and sex were from population-registry data and smoking and symptomatology was reported by questionnaire. (Symptomatic yes: symptomatic at the time of infection ascertained by positive PCR test; Symptomatic maybe: reported symptoms during the time period without ascertainment of specific time of infection by PCR test, with asymptomatic as reference meaning no symptoms during the time period until BL; Symptomatic no: no reported symptoms).

**Figure 2:**
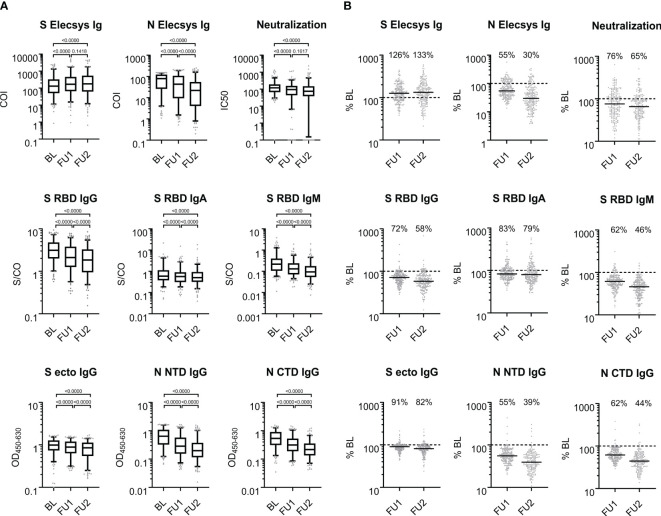
Antigen- and isotype-specific antibody reactivity over time. **(A)** SARS-CoV-2 reactive antibodies observed at baseline, first and second follow-up visit 5 and 10 months after baseline; BL, FU1 and FU2, respectively. For each of the three time points, the distribution of values is shown on a log10-scale in box plots (box: 1^st^ quartile, median and 3^rd^ quartile; whiskers 5^th^ and 95^th^ percentile; dots below and above the whiskers refer to points outside the 5^th^ and 95^th^ percentile). **(B)** Proportional antibody change comparing values at FU1 and FU2, respectively, to BL. % BL: For each of the follow-up time points, scatter plots of the individual changes in antibody reactivity as compared to BL are given for each measurement from **(A)**. The solid black line denotes the median change; the dashed line indicates 100% which means no change; the median change is stated as % for each follow-up time point. COI: cutoff index; S/CO: signal to cutoff ratio; OD_450-600_: optical density at 450 nm subtracted by the optical density at 630 nm; IC50: half maximal inhibitory concentration (fold serum dilution).

We analyzed the SARS-CoV-2 serum antibody levels in the 211 individuals with 13 serological tests in each individual at 3 time points (for abbreviations see **Table 2**): (i) 8 binding antibody tests (S RBD IgG, S RBD IgM, S RBD IgA, total Ig S [Elecsys S], N NTD IgG, N CTD IgG, total Ig N [Elecsys N]), (ii) 4 avidity tests for N (NTD and CTD) and S (RBD and S ecto), and (iii) a neutralization test. The raw data, median levels and p-values from Wilcoxon testing of the serum measurements are given in **Figure 2A** (see **Supplementary Table 1** for the respective tabular data of all measurements).

**Table 2:** Assays used in this study.

Abbreviation	Manufacturer	Antigen (domain)	Isotype	characteristic
Binding				
S Elecsys Ig	Roche	S (receptor-binding domain)	total Ig	quantitative Ig
N Elecsys Ig	Roche	N (unknown)	total Ig	quantitative Ig
S RBD IgG	in-house	S (receptor-binding domain)	IgG	quantitative IgG
S RBD IgA	in-house	S (receptor-binding domain)	IgA	quantitative IgA
S RBD IgM	in-house	S (receptor-binding domain)	IgM	quantitative IgM
S ecto IgG	in-house	S (ectodomain)	IgG	quantitative IgG
N NTD IgG	in-house	N (N-terminal domain)	IgG	quantitative IgG
N CTD IgG	in-house	N (C-terminal domain)	IgG	quantitative IgG
Neutralization				
Neutralization	in-house	S (complete)	total Ig	qualitative and quantitative Ig
Avidity				
S RBD IgG AI	in-house	S (receptor-binding domain)	IgG	qualitative, avidity
S ecto IgG AI	in-house	S (ectodomain)	IgG	qualitative, avidity
N NTD IgG AI	in-house	N (N-terminal domain)	IgG	qualitative, avidity
N CTD IgG AI	in-house	N (C-terminal domain)	IgG	qualitative, avidity

(S, spike protein; N, nucleocapsid protein; Ig, immunglobuline).

To assess the individual changes in antibody levels over time, we divided the antibody levels at FU1 or FU2 by the corresponding BL levels (in percent, %BL). The medians of the %BL values indicate waning antibody levels for all binding assays except for the S-Elecsys Ig ECLIA test, tested by the Wilcoxon signed-rank test and judged at the 1% significance level, for at least one of the FUs (**Figure 2B**). Interestingly, a waning of the serum neutralization capacity between FU1 and FU2 was not observed (p=0.1617). Furthermore, the S Elecsys Ig ECLIA showed a significant increase (p<0.01) at FU1 and FU2 compared to BL.

**Figure 3:**
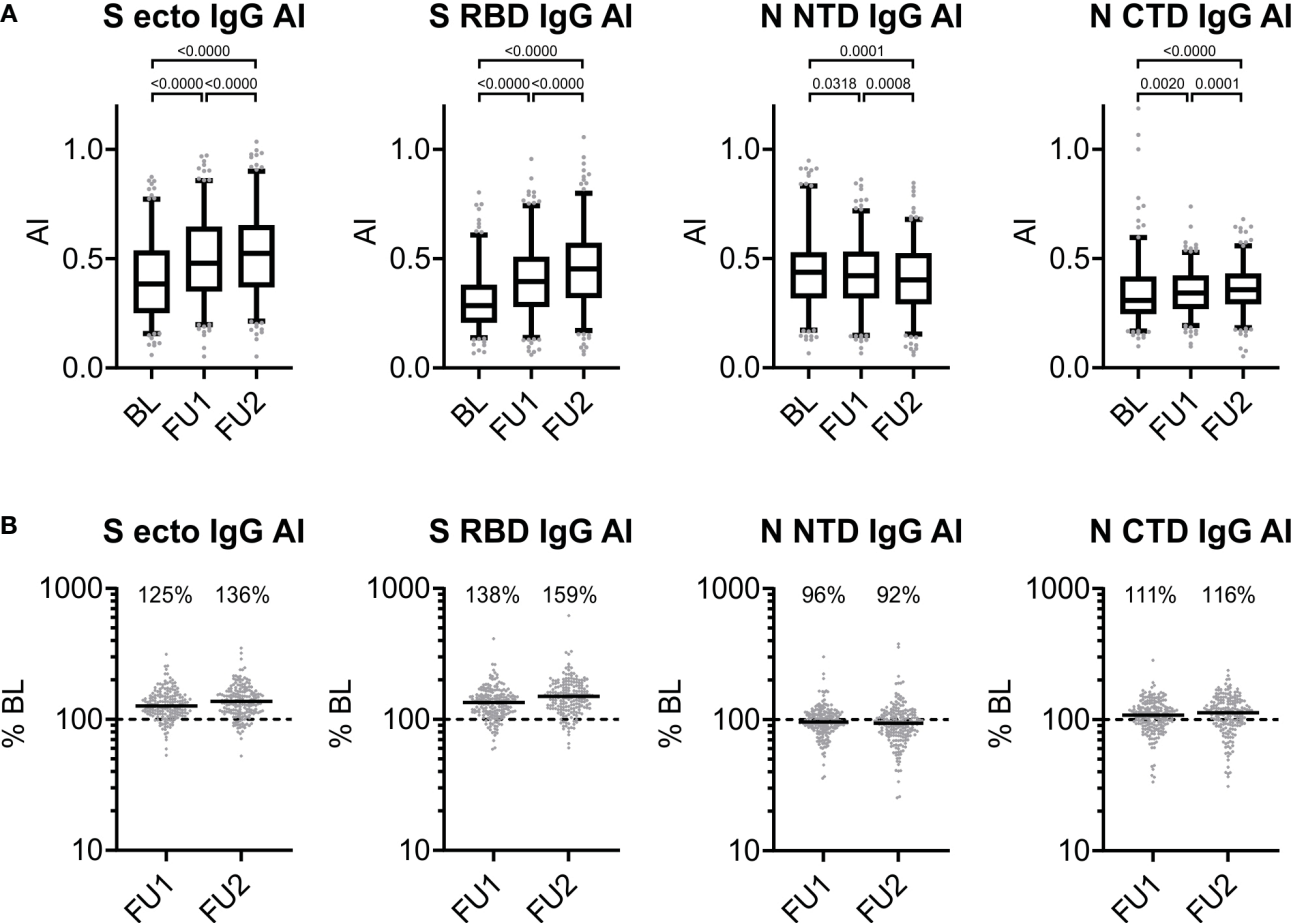
Antigen-specific changes of avidity over time. **(A)** Avidity index (AI) of serum IgG directed against S ecto, S RBD, N NTD, and N CTD observed at baseline, first and second follow-up visit 5 and 10 months after baseline (BL, FU1 and FU2, respectively): for each of the three time points, we show the distribution of values (box: 1^st^ quartile, median and 3^rd^ quartile; whiskers 5^th^ and 95^th^ percentile; dots below and above the whiskers refer to observed values outside the 5^th^ and 95^th^ percentile) and p-values from Wilcoxon signed-rank test. **(B)** Proportional changes in the AI at first or second follow-up (FU1, FU2) compared to BL (in percent). For each of the three time points, we show individual changes in avidity at FU1 and FU2 compared to BL (% BL). The dashed line represents the 100% BL level (i.e. no change), horizontal solid lines show the median values, and the median change is stated as % for each follow-up time point compared to BL.

We hypothesized that the antibody-antigen-bridging principle of the ECLIA test was more sensitive for the affinity maturation of the serum antibodies and that the S-specific antibodies were subjected to a more distinct affinity maturation as compared to the N-protein-directed antibodies. To answer this, we applied our ELISA to determine the avidity of the sera of the 211 individuals at each of the three time points. Consistent with our hypothesis, the mean increase in avidity over time was more pronounced for the S-based assays, S RBD IgG and S ecto IgG, than for the N-based assays: higher increase to 125% for FU1 and 136% for FU2 for S ecto IgG AI and 138% for FU1 and 159% for FU2 for S RBD IgG AI as compared to decrease to 96% for FU1 and 92% for FU2 for N NTD IgG AI and increase to 111% for FU1 and 116% for FU2 for N CTD IgG AI (all changes significant with p<0.01, except the decrease to 96% for FU1 for N NTD IgG; see **Figure 3**). Interestingly, we found that S-directed affinity maturation continued for at least > 8 months post infection as measured AIs still increased from FU1 to FU2.

**Figure 4:**
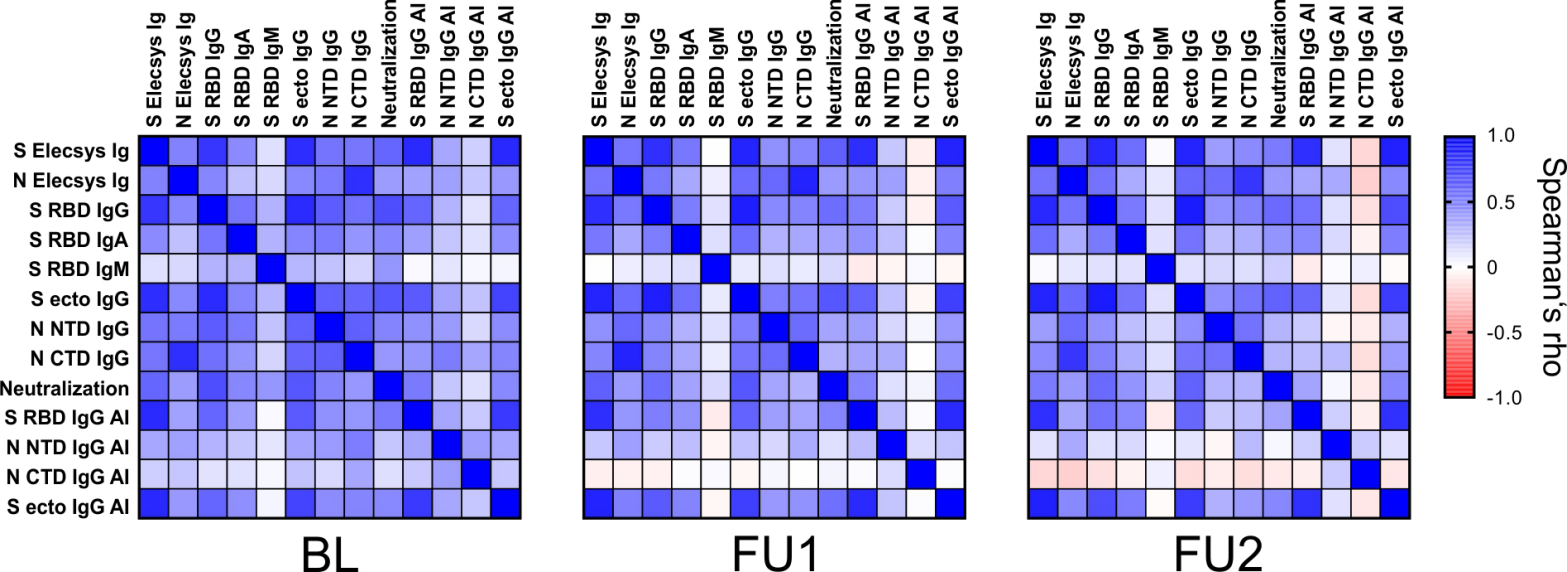
Correlation of the different serological parameters at the three visits. Spearman’s rho values of pairwise comparisons between the test results are plotted for the three visits BL, FU1 and FU2. A color map for the gradient of values is given on the right, ranging from Spearman’s rho values from –1 to 1. (Spearman’s rho values and corresponding p-values are given in **Supplementary Table 2**).

To assess the relationship between the different assays, we generated correlation matrices for each of the three visits based on nonparametric, rank-based correlations (**Figure 4**, **Supplementary Table 2**). For all parameters, a trend towards weaker between-assay correlation was observed over time, but the general pattern is maintained. As expected, test results based on the same antigen generally showed a strong correlation. Strongest correlations (Spearman’s rho, ρ) at BL were observed for S RBD IgG vs. S ecto IgG (ρ = 0.83), S ecto IgG vs. S Elecsys Ig (ρ = 0.82) and S RBD IgG vs. S Elecsys Ig (ρ = 0.79) at an alpha level of <0.0001. In line with our earlier findings (41), best correlations with the pseudotype neutralization were observed for S RBD IgG (ρ = 0.69 at BL) and S ecto IgG (ρ = 0.66 at BL). N-based assay results were only moderately correlated to the pseudotype neutralization test results (at BL: N NTD IgG, ρ = 0.48; N CTD IgG, ρ = 0.41; N Elecsys Ig, ρ = 0.38). The correlation of the Elecsys N Ig test with the C-terminal domain was stronger (ρ = 0.81) as compared to the N-terminal domain (ρ = 0.52). Assuming that the complete N antigen is used in the Elecsys N Ig test (no information about the antigen available from the manufacturer), this indicates a bias in the humoral immunogenicity towards the C-terminal domain. Interestingly, avidity indices (AI) against S RBD and S ecto were strongly correlated to the serum reactivities in the Elecsys S Ig test (ρ = 0.83 and 0.84, respectively). This further supports our hypothesis of a strong weighting of affinity maturation in the ECLIA bridging test. S-based AIs correlate moderately with Neutralization (S RBD: ρ = 0.52; S ecto: ρ = 0.48), while N-based AIs are not correlated with Neutralization (N NTD: ρ = 0.22; N-CTD: ρ = 0.13). Finally, IgM-type antibodies directed against S-RBD (S RBD IgM) showed the lowest correlation with all other serum parameters. This is probably due to the generally rapid waning of IgM and thus low levels of IgM measured at all visits.

Next, we investigated whether age, sex, or smoking status had an effect on titer levels or titer change over time for any of the 13 assays. For this, we employed one LMM for each of the 13 titer levels as outcome variable, with the two time-variables (timeFU1, timeFU2), age, sex, and smoking as well as their interactions with the time variables as covariates, in order to analyze the 211 individuals and 633 measurements for each respective assay. We judged significance of the association of covariates with each of the 13 antibody levels at the 1% significance level.

First of all, we found higher BL levels by older age for all 13 antibody/avidity assays (i.e. positive coefficient for the covariate “age”; **Table 3**, **Supplementary Table 3**): for most assays, this was highly significant (P_age_<0.001) and significant for S RBD IgG AI (P_age_=0.008); coefficients for age were positive, but not significant for N Elecsys Ig, S RBD IgM, N NTD IgG AI, N CTD IgG AI, or S ecto IgG AI (P_age_=0.066, 0.014, 0.012, 0.332, 0.028, respectively). For example, N Elecsys Ig levels were ~1% higher for 1 additional year of age (exp(coefficient)=exp(0.010)=1.010; **Table 3**) or 49% higher for 40 additional years of age (exp(40*coefficient)=exp(40*0.010)=1.492).

**Table 3:** Association of age, sex, and smoking on five selected antibody and avidity levels and level change from linear mixed models.

	Association with level coefficient (s.e.); P	Association with change until FU1	Association with change until FU2
S Elecsys Ig			
intercept/time point	5.186 (s.e.: 0.156; p<0.001)	0.073 (s.e.: 0.069; p: 0.291)	0.078 (s.e.: 0.07; p: 0.262)
age	0.021 (s.e.: 0.006; p<0.001)	-0.001 (s.e.: 0.002; p: 0.736)	-0.003 (s.e.: 0.002; p: 0.234)
sex	-0.283 (s.e.: 0.194; p: 0.144)	0.253 (s.e.: 0.083; p: 0.003)	0.279 (s.e.: 0.084; p: 0.001)
smoker_current	-0.628 (s.e.: 0.236; p: 0.008)	-0.069 (s.e.: 0.151; p: 0.645)	-0.17 (s.e.: 0.146; p: 0.247)
smoker_ex	-0.176 (s.e.: 0.188; p: 0.349)	0.214 (s.e.: 0.101; p: 0.035)	0.324 (s.e.: 0.102; p: 0.002)
N Elecsys Ig			
intercept/time point	4.035 (s.e.: 0.158; p<0.001)	-0.448 (s.e.: 0.08; p<0.001)	-1.149 (s.e.: 0.081; p<0.001)
age	0.01 (s.e.: 0.006; p: 0.066)	0.015 (s.e.: 0.003; p<0.001)	0.021 (s.e.: 0.003; p<0.001)
sex	-0.1 (s.e.: 0.193; p: 0.604)	0.049 (s.e.: 0.096; p: 0.613)	0.287 (s.e.: 0.097; p: 0.003)
smoker_current	-0.528 (s.e.: 0.253; p: 0.038)	-0.179 (s.e.: 0.174; p: 0.305)	-0.424 (s.e.: 0.169; p: 0.012)
smoker_ex	-0.069 (s.e.: 0.197; p: 0.725)	-0.037 (s.e.: 0.117; p: 0.748)	-0.094 (s.e.: 0.118; p: 0.426)
S RBD IgG			
intercept/time point	1.265 (s.e.: 0.075; p<0.001)	-0.462 (s.e.: 0.04; p<0.001)	-0.658 (s.e.: 0.04; p<0.001)
age	0.013 (s.e.: 0.003; p<0.001)	0.001 (s.e.: 0.001; p: 0.359)	-0.001 (s.e.: 0.001; p: 0.464)
sex	-0.104 (s.e.: 0.091; p: 0.258)	0.13 (s.e.: 0.047; p: 0.006)	0.166 (s.e.: 0.048; p: 0.001)
smoker_current	-0.402 (s.e.: 0.122; p: 0.001)	0.054 (s.e.: 0.086; p: 0.527)	-0.027 (s.e.: 0.083; p: 0.744)
smoker_ex	-0.111 (s.e.: 0.094; p: 0.239)	0.112 (s.e.: 0.057; p: 0.051)	0.135 (s.e.: 0.058; p: 0.021)
Neutralization			
intercept/time point	4.943 (s.e.: 0.123; p<0.001)	-0.446 (s.e.: 0.121; p<0.001)	-0.594 (s.e.: 0.123; p<0.001)
age	0.02 (s.e.: 0.004; p<0.001)	-0.005 (s.e.: 0.004; p: 0.216)	0.002 (s.e.: 0.004; p: 0.617)
sex	-0.026 (s.e.: 0.148; p: 0.861)	0.034 (s.e.: 0.146; p: 0.813)	0.243 (s.e.: 0.147; p: 0.1)
smoker_current	-0.55 (s.e.: 0.249; p: 0.028)	0.286 (s.e.: 0.263; p: 0.277)	-0.009 (s.e.: 0.258; p: 0.974)
smoker_ex	-0.212 (s.e.: 0.176; p: 0.227)	0.11 (s.e.: 0.177; p: 0.535)	-0.04 (s.e.: 0.179; p: 0.824)
S RBD IgG AI			
intercept/time point	-1.198 (s.e.: 0.054; p<0.001)	0.249 (s.e.: 0.033; p<0.001)	0.357 (s.e.: 0.033; p<0.001)
age	0.005 (s.e.: 0.002; p: 0.008)	-0.002 (s.e.: 0.001; p: 0.04)	-0.004 (s.e.: 0.001; p: 0.002)
sex	-0.062 (s.e.: 0.066; p: 0.344)	0.083 (s.e.: 0.04; p: 0.036)	0.031 (s.e.: 0.04; p: 0.434)
smoker_current	-0.073 (s.e.: 0.093; p: 0.433)	-0.052 (s.e.: 0.072; p: 0.467)	-0.009 (s.e.: 0.07; p: 0.894)
smoker_ex	-0.055 (s.e.: 0.071; p: 0.435)	-0.059 (s.e.: 0.049; p: 0.225)	0.026 (s.e.: 0.049; p: 0.597)

Results from five linear mixed models (LMMs), one for each of the following five selected antibody and avidity levels as outcome: S Elecsys Ig, N Elecsys Ig, S RBD IgG ELISA, pseudotype neutralization and S RBD IgG-based determination of the avidity index (ln-transformed to ensure approximate normal distribution). The covariates were 2 time-variables (timeFU1, timeFU2) and age (centered at 50 years), sex (1=women, 0=men), and 2 smoking variables (current smoking, ex-smoking versus never smokers as reference) as well as their interactions with the time-variables. For each of age, sex, and smoking variables, we show the covariate estimates and standard errors (s.e.) on the natural logarithm of titer levels (1^st^ column) and on titer change between BL and FU1 or FU2 (2^nd^ column or 3^rd^ column, respectively; i.e. from the interaction terms with timeFU1 or timeFU2). In the first row per outcome, we show the intercept (1^st^ column) and the association of timeFU1 or timeFU2 (2^nd^ or 3^rd^ column, respectively). The analogous results for the remaining 8 assay levels are shown in **Supplementary Table 3**.

Next, we evaluated the associations of timeFU1 or timeFU2 with titer levels in the LMM, which can be interpreted as titer change between BL and FU1 or FU2, respectively. Whenever the timeFU1 or timeFU2 associations on titer level were not significant, a significant interaction of age, sex, or smoking with timeFU1 or timeFU2 is also to be interpreted as significant change over time – at least in a subgroup of individuals with certain age, sex, or smoking status.

In line with the Wilcoxon signed-rank test results, there was a significant change for all 13 assays (P_timeFU1_, P_timeFU1_< 0.01; except for S Elecsys Ig, P_timeFU1 =_ 0.291 and P_timeFU1 =_ 0.262, but P_sex*timeFU2_<0.01 indicating a significant change over time in women, but not in men; see **Table 3**, **Supplementary Table 3**). For example for N Elecsys Ig, we found a drop down to 63.9% until FU1 and to 31.7% until FU2 (exp(-0.448)=0.639 or exp(-1.149)=0.317, respectively).

The LMM also allowed for evaluating the interaction of age with timeFU1 or timeFU2 on titer levels, which can be interpreted as the age-effect on titer change: since all 13 assays showed higher BL-levels by higher age (see above), interaction effects age*timeFU1 or age*timeFU2 into the same direction as the timeFU1 or timeFU2 effects indicate a stronger titer change for older individuals, opposite directions indicate a smaller titer change.

Most of the 13 titer levels showed a significant change over time with interesting patterns (**Table 3** for 5 assays of primary interest capturing different biological components; **Supplementary Table 3** for the other 8 assays):

For the 9 antibody and neutralization tests, we found that

(1) Most antibodies and serum neutralization showed a decline over time (all except S Elecsys Ig) with three distinct patterns how age modulated this decline: (i) higher age reduced the decline: N Elecsys Ig (P_age*timeFU1_ and P_age*timeFU2_<0.001), i.e. N Elecsys Ig levels decreased less for older individuals; (ii) age had no effect on decline: S RBD IgG, Neutralization, N NTD IgG, N CTD IgG, S ecto IgG (P_age*timeFU1_ and P_age*timeFU2_>0.211), i.e. these titer levels decreased irrespective of age; (iii) higher age reinforced the decline: S RBD IgA and S RBD IgM (P_age*timeFU1_ or P_age*timeFU2_<0.001), i.e. older individuals lost S RBD IgA and S RBD IgM antibody levels more quickly than younger individuals;
(2) One antibody showed increase over time: S Elecsys Ig increased over time in women, but not in men; age had no effect on the increase (P_age*timeFU1_ and P_age*timeFU1 =_ 0.736 and 0.234).

For the 4 avidity assays, we found:

(3) Avidity declined over time: N NTD IgG AI (P_age*timeFU1_ and/or P_age*timeFU2_<0.001); age had no effect on decline (P_age*timeFU1_ and P_age*timeFU2 =_ 0.944 and 0.075);
(4) Avidity increased over time: (i) higher age reduced the increase: S RBD IgG AI (P_age*timeFU2 =_ 0.002); (ii) age had no effect on the increase: S ecto IgG AI, N CTD IgG AI (P_age*timeFU1_ and P_age*timeFU2_>0.176).

**Figure 5:**
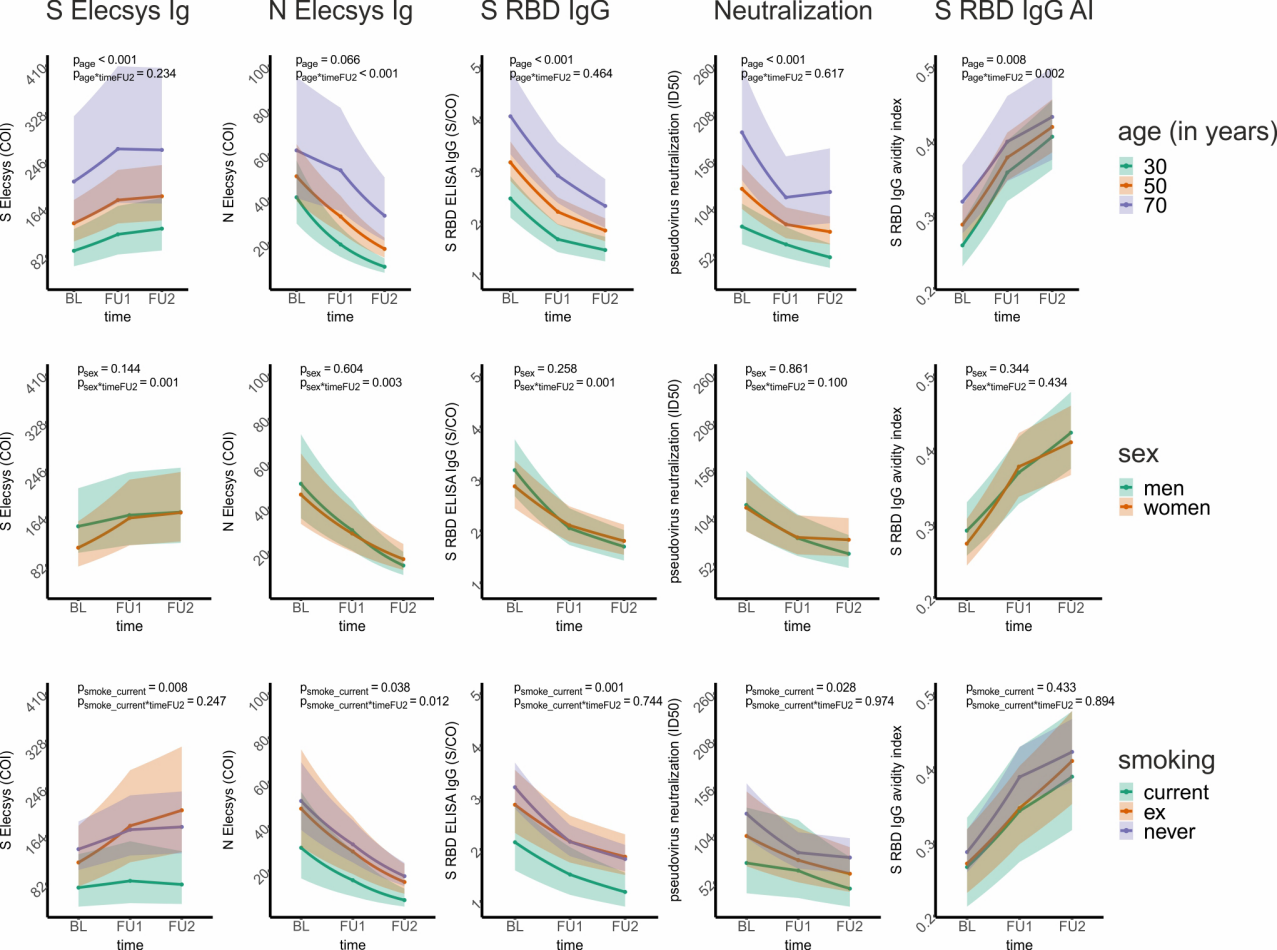
Graphical presentation of the association of age, sex, and smoking on five selected antibody and avidity levels and change over time from linear mixed models. For five selected antibody and avidity levels – S Elecsys Ig, N Elecsys Ig, S RBD ELISA IgG, pseudotype neutralization, and S RBD IgG-based determination of the avidity index – we show the effect of age, sex, and smoking status on levels and level change. For this, a linear mixed model (LMM) was employed for each of the 5 outcome variables (i.e., levels; ln-transformed to ensure approximate normal distribution), with both time variables (timeFU1, timeFU2), age (centered at 50 years), sex, and smoking status (current, ex-, and never-smoker) as well as their interactions with the time variables as covariates. The model-based predicted effects for each of the 5 assay outcomes (re-transformed using the exponential function and thus on the original scale) are shown for several example individuals (first row: 30-, 50-, and 70-year-old individuals; second row: men and women; third row: current, ex-, and never-smokers). The shaded regions denote the corresponding 99% confidence intervals. We state the p-value of the respective covariate association on levels and level change until FU2 in each panel, while all estimates and P-values for these models are shown in **Table 3**.

To understand the extent of the effects of age on titer levels and change, we visualized the LMM-predicted titer levels over time for individuals aged 30, 50, and 70 years. This demonstrates the substantial impact of age on titer levels at baseline as well as follow-up assessments with visible differentiation of the 99%-confidence intervals (**Figure 5**; **Supplementary Figure 1**).

When evaluating the effect of sex or smoking status on titer levels and titer change in our LMM analyses (i.e. looking at coefficients and P-values for sex and smoking status as well as their interaction with timeFU1 and timeFU2), we found (**Table 3**, **Supplementary Table 3**): (i) antibody decline was stronger in men than in women for multiple assays (S RBD IgG, S RBD IgM, S ecto IgG, N Elecsys Ig, N NTD IgG, N CTD IgG, N NTD IgG AI, P_sex*timeFU2_<0.01 and positive), while there was no significant sex-effect for any type of antibody levels or avidities at baseline (i.e. P_sex_>0.01). (ii) Current smokers had lower binding levels (S RBD IgG, S Elecsys Ig, S ecto IgG; P_smoker_current_=0.001, 0.008, 0.004) and decreased avidity levels (AI S ecto, P_smoker_current_=0.007) compared to never-smokers. There was no evidence for differential change of the measured titers over time between current and never smokers for any assay (P_smoker_current*timeFU1_ and P_smoker_current*timeFU2_ ≥ 0.01). When visualizing the LMM-predicted titer levels over time by sex and by smoking status, we found that the abovementioned sex associations with titer change were very small and mostly due to men (as compared to women) having slightly higher titer levels at BL and similar levels at FU1 and FU2 (S Elecsys Ig, S RBD IgG: **Figure 5**; S IgM, NCTD-IgG, S ecto IgG, **Supplementary Figure 1**). The associations for current smokers versus never smokers on titer levels for S Elecsys Ig and S RBD IgG were visible with lower titer levels also at FU1 and FU2 (**Figure 5**; also for S ecto IgG and ecto IgG AI, **Supplementary Figure 1**).

When comparing the LMM results with and without including smoking as a covariate, we found no major differences in the coefficients for timeFU1, timeFU2, age, sex, or their interaction (**Supplementary Table 4**).

It was striking that all antibody titers at baseline and during follow-up were higher the older the individuals were. A potential explanation for this is a longer or more severe disease at higher age, for which more severe COVID-19 symptoms can be an indicator. We explored this by evaluating whether symptoms (“symptomatic” infected, “maybe symptomatic” infected, or “asymptomatic” infected, **Table 1**) reported in the BL questionnaire had an association on titer levels at BL or altered the association of age with BL titer levels (i.e., interaction of age with symptomatic variables) using an extended LMM. Since this analysis was exploratory, we interpreted results as noteworthy when (i) the average titer level was higher for symptomatic than for asymptomatic individuals at a less strict P-value<0.10 and/or (ii) the symptom-age interaction coefficient was at least as large as the coefficient of the age main effect, again with P-value<0.10.

Regarding average assay levels, we found 6 out of all 13 assays to be noteworthy (**Table 4**, **Supplementary Table 5**). For Neutralization, for instance, a 50-year old with symptoms – as compared to a 50-year old asymptomatic – had a 51% higher titer level at BL (exp(0.414)=1.51), or, for N Elecsys Ig, a 124% higher level (exp(0.808)=2.24). For eight of the 13 assays, we found a noteworthy interaction of symptoms and age on titer levels, that is, symptomatology changed the association between age and titer levels (**Table 4**, **Supplementary Table 5**). For example for S RBD IgG or N Elecsys Ig, there was barely any age-association with titer levels among asymptomatic (i.e. age coefficient near zero) and a predominant age-association only among the symptomatic_yes (i.e. large symptomatic_yes*age interaction coefficient): the average S RBD IgG titer levels for asymptomatic individuals were hardly affected by age (exp(0.006)=1.006, so an average increase of only 0.6% per year of age), while the age effect was substantial for symptomatic subjects (exp(0.006 + 0.018)=1.024, meaning an average increase of 2.4% per year of age); similar for N Elecsys Ig.

**Table 4:** Association of symptomology and its interaction with age on five selected antibody and avidity levels using linear mixed models.

	Association with level coefficient (s.e.); P	Interaction with age coefficient (s.e.); P
S Elecsys Ig		
age	0.022 (s.e.: 0.013; p: 0.102)	–
symptomatic_yes	0.450 (s.e.: 0.315; p: 0.154)	0.034 (s.e.: 0.018; p: 0.058)
symptomatic_maybe	-0.038 (s.e.: 0.272; p: 0.888)	-0.015 (s.e.: 0.015; p: 0.32)
N Elecsys Ig		
age	0.008 (s.e.: 0.013; p: 0.556)	–
symptomatic_yes	0.808 (s.e.: 0.312; p: 0.01)	0.032 (s.e.: 0.017; p: 0.066)
symptomatic_maybe	0.617 (s.e.: 0.269; p: 0.022)	-0.007 (s.e.: 0.015; p: 0.635)
S RBD IgG		
age	0.006 (s.e.: 0.006; p: 0.377)	–
symptomatic_yes	0.236 (s.e.: 0.149; p: 0.115)	0.018 (s.e.: 0.008; p: 0.027)
symptomatic_maybe	0.053 (s.e.: 0.129; p: 0.679)	0.004 (s.e.: 0.007; p: 0.567)
Neutralization		
age	0.020 (s.e.: 0.009; p: 0.035)	–
symptomatic_yes	0.414 (s.e.: 0.211; p: 0.05)	0.014 (s.e.: 0.012; p: 0.236)
symptomatic_maybe	0.351 (s.e.: 0.183; p: 0.055)	-0.004 (s.e.: 0.01; p: 0.693)
S RBD IgG AI		
age	0.006 (s.e.: 0.004; p: 0.154)	–
symptomatic_yes	0.046 (s.e.: 0.103; p: 0.659)	0.010 (s.e.: 0.006; p: 0.094)
symptomatic_maybe	-0.115 (s.e.: 0.089; p: 0.195)	-0.006 (s.e.: 0.005; p: 0.244)

Results from five linear mixed models (LMMs), one for each of the following five antibody and avidity levels as outcome: S Elecsys Ig, N Elecsys Ig, S RBD IgG ELISA, pseudotype neutralization and S RBD IgG-based determination of the avidity index (ln-transformed). These results are based on an extension of the main model (see **Table 3**) by adding 2 symptomology variables (symptomatic_yes=symptomatic at the time of infection ascertained by positive PCR test, symptomatic_maybe=reported symptoms during the time period without ascertainment of specific time of infection by PCR test, with asymptomatic as reference meaning no symptoms during the time period until BL) and their age-interaction. This table shows covariate estimates, standard errors (s.e.) and P-values for the associations of the symptomology variables with outcomes (1^st^ column) and their interaction with age (2^nd^ column; age centered at 50 years). The 1^st^ row indicates the association of age with respective BL titer level among asymptomatic individuals (reference group); the 2^nd^ and 3^rd^ row indicates the association of being symptomatic_yes or symptomatic_maybe, respectively, versus asymptomatic (1^st^ column) and whether there was a differential age-association among symptomatic_yes or symptomatic_maybe (i.e. interaction with age) compared to the age-association among asymptomatic. The analogous results for the remaining 8 titer levels are shown in **Supplementary Table 5**.

Our results suggest that the association of higher age with higher antibody levels is found predominantly among individuals with symptomatic infection, but hardly among asymptomatic infected individuals.

## 4 Discussion

We analyzed the levels and temporal course of SARS-CoV-2 reactive antibodies in the 211 unvaccinated participants of the TiKoCo study who were SARS-CoV-2 seropositive at baseline, had attended two follow-up visits at 5 and 10 months after the first sampling (8 and 13 month after infection), and had not been vaccinated during the observation period. Using 8 different S- and N-binding assays and a pseudotype neutralization test, we found a decline of S- and N-specific antibody levels as well as of pseudotype neutralization over one year after infection in all but one avidity biased assay. The waning of N-specific antibody levels was more pronounced than the S-specific. However, our results suggest a substantial decline of neutralization-based protection one year after infection. Whether the different antibody levels continue to decrease or have reached a plateau and how long the avidity continues to increase cannot be answered with our data set and therefore requires further investigation.

Similar to other population-based studies (16, 20, 52), we found significantly higher antibody levels after infection for older individuals compared to younger for most binding assays. This difference by age vanished when excluding individuals with more symptomatic infections. This is in line with a hypothesis that, for older individuals, both higher antibody levels and more symptoms are due to higher disease severity at older age. In contrast, lower antibody levels have been reported in older individuals after vaccination (52). If this is a result of different immunological processes during vaccination compared to natural infection is currently unclear. Furthermore, and not yet described, IgA and IgM antibody levels decline more quickly in elderly individuals. Interestingly, the higher antibody BL-levels for individuals of older age generally did not decline down to the levels of younger individuals over the course of the one year after infection that our data covered. There was no sex-difference in antibody levels, but some more pronounced antibody decline for men compared to women. This population-level sex-difference was small, but would be in line with sex-differences in the humoral immune response to infection as well as to vaccination as described previously (53–55).

We found current smokers to exhibit lower antibody levels than non-smokers for some assays (S RBD IgG, S Elecsys Ig, S ecto IgG, AI S ecto), but comparable antibody decline. While this difference in titer levels by smoking status was observed among these 211 seropositive individuals analyzed here, we had previously found no difference by smoking status when analyzing 237 individuals with registered, PCR-test confirmed infections (56). This finding is in line with a lack of antibody level difference by smoking status among mostly symptomatic (and thus PCR-tested) individuals, but lower antibody levels among smokers when asymptomatic individuals are included.

S-specific serum avidity increased markedly over time throughout the study period of 1 year after infection, in contrast to N-specific avidity, which showed a minor decrease for N-NTD and a slight increase for N-CTD. This has been consistently described by others (21, 27, 57, 58). Interestingly, an increase was also observed in S-specific antibody levels measured in an affinity-sensitive assay (Roche Elecsys S Ig). Our results are in line with an increased avidity counter-acting the decreased antibody quantity for S-specific antibodies, depending on the architecture of the assay. Avidity increase does not compensate decrease in titer with regards to neutralization.

The population-based longitudinal design of our study defines infection by serostatus rather than relying on registered infections. An advantage here is the inclusion of asymptomatically infected individuals in a proportion that reflects the general population. Most of the 211 seropositive participants (181; 86%) had no PCR- and/or antigen-test-confirmed SARS-CoV-2 infection (10). However, 137 (65%) of the seropositives reported symptoms that could have been suggestive of SARS-CoV-2 infection. The high number of subjects who were unaware of their infection is likely due to low testing capacity of SARS-CoV-2 infections available at this early stage of the pandemic and the inclusion of individuals as young as 14 years of age. We might have misclassified some symptomatic individuals into the asymptomatic group, as we did not include “sore throat” or “diarrhea” as symptoms, both of which were considered SARS-CoV-2 related (59), but also to lack specificity for SARS-CoV-2 infection in a general population. Also, we cannot exclude that some of the asymptomatic individuals might have been (mis)classified as “maybe symptomatic”, since the exact date of infection was unknown and the reported symptoms during the time period were in part not specific. Thus, we acknowledge a certain level of potential misclassification of the symptomatology variables. Still, we asked for the symptoms before the individual was informed about being seropositive or seronegative, avoiding a recall bias.

Another advantage of the present study is the relatively short period of time during which the infections occurred. Using the registered cases and an incubation period of about 6 days (60), a period of about 4 weeks can be defined, which lasted from the last week of February until about one week after the start of the lockdown on March 18 (44), although due to the high number of asymptomatic infections, individual earlier infections cannot be excluded. This results in a time-since-infection of approximately 3 months for the baseline sera analyzed here and thus a follow-up at about 8 and 13 months after infection. Several studies have described nearly complete seroconversion after 14 days, with variations depending on antibody isotype, and the peak of initial antibody response at approximately 1-3 months post infection (22, 24, 45, 61–63). Thus, our baseline sampling took place after seroconversion, B cell expansion, and peak serum antibody levels. Another advantage is that vaccination had just been introduced shortly before our 2^nd^ follow-up and thus most individuals in our cohort had been unvaccinated across all three time points and the few already vaccinated at the 2^nd^ follow-up were excluded in our analyses here.

Various studies have shown substantial variability between the quantitative outcomes of SARS-CoV-2 serological tests. We thus employed multiple assays with different antigens and assay architecture to recognize assay-dependent bias (32, 64). The commercial assay we used is based on a sandwich assay principle that detects the bridging of two differently modified monomeric antigens by the two (or more in case of IgM) paratopes of an antibody (bridging ECLIA). Thus, multivalent antigen binding, which means less affine binding to multiple antigens, is impossible and the assay is strongly dependent on the affinity of the serum Ig against the antigen. In contrast, ELISA-based measurements are sensitive for multivalent Ig-antigen interactions. Further, the pseudotype neutralization test is also sensitive for such avidity effects, as the antigens are densely arrayed on the surface of the virus-like particles. This likely explains the generally higher correlation of pseudotype neutralization with ELISA rather than the commercial bridging ECLIA at BL (i.e. at the beginning of affinity maturation) and a better reflection of the course of the neutralizing antibody levels by the ELISA over time. It is consistent with Stone et al., who described increasing antibody levels for four S-directed total Ig bridging assays, but waning levels for all other assays tested in their panel (64).

In our data, S-based antibody binding assays correlate well with pseudotype virus neutralization, which is a widely accepted correlate of protection against SARS-CoV-2 infection (1, 65). Thus, our results suggest a significant decline of neutralization-based protection one year after infection. Nevertheless, we found raising avidity levels until the end of our observation time, which was up to 13 months after infection, leading to optimized binding of neutralizing antibodies even at lower concentrations. Efficient binding even at low antigen concentrations can be crucial, especially in case of a local infection in the respiratory tract. Since convalescent subjects have been shown to develop higher avidity after booster vaccination than dually vaccinated subjects, it can be assumed that prolonged contact with the antigen and therefore improved maturation of B cells is important (66). This supports the relevance of multiple booster vaccinations separated by sufficiently long periods for affinity maturation or even novel vaccine formulation strategies providing extended antigen availability.

As we use similar assay protocols for the detection of different antigen-specific antibodies, a comparison between the different assay results is possible. We observed a divergent antigen-specific course of antibody levels over time for the N- and S-protein. From our data, we can conclude that N-specific antibody serum levels are less durable over time than S-specific levels. This has consistently been described by others (20, 27). Here, we can provide additional evidence at the population level and with up to antigen domain-specific resolution of the response. Our findings are in-line with waning levels of S-specific serum antibodies and immunity after infection (67–72) and after vaccination with the currently licensed vaccines (46, 48, 58, 73–76). However, head-to-head comparative studies on efficacy and durability of antibody levels and the corresponding effector functions of S- and N-based vaccines are currently missing.

It can be considered a limitation that our serological analyses are based on the SARS-CoV-2 Wuhan strain and that no data on VOC virus variants is shown. As the sampling took place in the early phase of the pandemic, where no VOCs were circulating, we sought to focus on the autologous response and its stability. Further, the two employed commercial assays were also based on the wild type sequence. However, it should be emphasized that studies on N- or S-specific serological response specifically after isolated infections are now hardly possible due to interference by the ongoing vaccination campaigns and multiple infections per person. Our study can thus serves as a model that avoids interference by vaccination and can investigate the temporal course after an isolated infection. Evaluating antibody response after multiple infections by various VOCs on top of multiple vaccinations in population-based settings will be highly challenging.

In summary, our longitudinal analyses of a comprehensive set of 13 SARS-CoV-2 antibodies assays helps understand the temporal course of SARS-CoV-2 antibody quantity and quality. This is highly relevant as it shapes our knowledge on limitations of the concept of herd immunity, informs future decisions on the characteristics of vulnerable groups and shows strengths and limitations of specific test strategies for protective immunity.

## Data availability statement

The raw data supporting the conclusions of this article will be made available by the authors, without undue reservation.

## Ethics statement

The studies involving humans were approved by Ethics Committee of the University of Regensburg (vote 20-1867-101) and the Ethics Committee of the University of Erlangen (vote 248_20 Bc). The studies were conducted in accordance with the local legislation and institutional requirements. Written informed consent for participation in this study was provided by the participants’ legal guardians/next of kin.

## Author contributions

RW and KÜ conceived the study. RW, KÜ, IH, OG, and DP conceptualized the study. RW, IH, RB, and DP supervised the research activities. HN, SB, PS collected the blood samples. DP developed the serological methodology. DP, SE, and AP collected the data. Data curation was done by SW, DP, FG, and SE. DP, SW and IH performed the statistical analysis. Visualization of the data was done by DP and SW. DP drafted the manuscript. SW, IH and RW contributed to the revision of the manuscript. All authors contributed to the article and approved the submitted version.
