# Supplementary material for: Population-based study of the durability of humoral immunity after SARS-CoV-2 infection
Source: Front Immunol. 2023 Oct 5;14:1242536. doi: 10.3389/fimmu.2023.1242536 (PMC10585261; doi:10.3389/fimmu.2023.1242536)
Supplement: Supplementary file 1 [file DataSheet_1.pdf]

Supplementary Material

Population-based study of the durability of humoral immunity after SARS-CoV-2 infection

Supplementary Figures

Supplementary Figure 1: Graphical presentation of the association of age, sex, and smoking on eight antibody and avidity levels and change over time from linear mixed models. For eight antibody and avidity levels - S RBD ELISA IgA, S RBD ELISA IgM, N NTD ELISA IgG, N CTD ELISA IgG, N NTD IgG AI, N CTD IgG AI, S ecto ELISA IgG, and S ecto ELISA IgG AI - we show the effect of age, sex, and smoking status on levels and level change. For this, a linear mixed model (LMM) was employed for each of the 8 outcome variables (i.e., levels; ln-transformed to ensure approximate normal distribution), with both time variables (timeFU1, timeFU2), age, sex, and smoking status (current, ex-, and never-smoker) as well as their interactions with the time variables as covariates. The model-based predicted effects for each of the 8 assay outcomes (re-transformed using the exponential function and thus on the original scale) are shown for several example individuals (first row: 30-, 50-, and 70-year-old individuals; second row: men and women; third row: current, ex-, and never-smokers). The shaded regions denote the corresponding 99% confidence intervals. The coefficients and their standard errors for each model are shown in Supplementary Table 4.

Supplementary Material: Population-based study of the durability of humoral immunity after SARS-CoV-2 infection

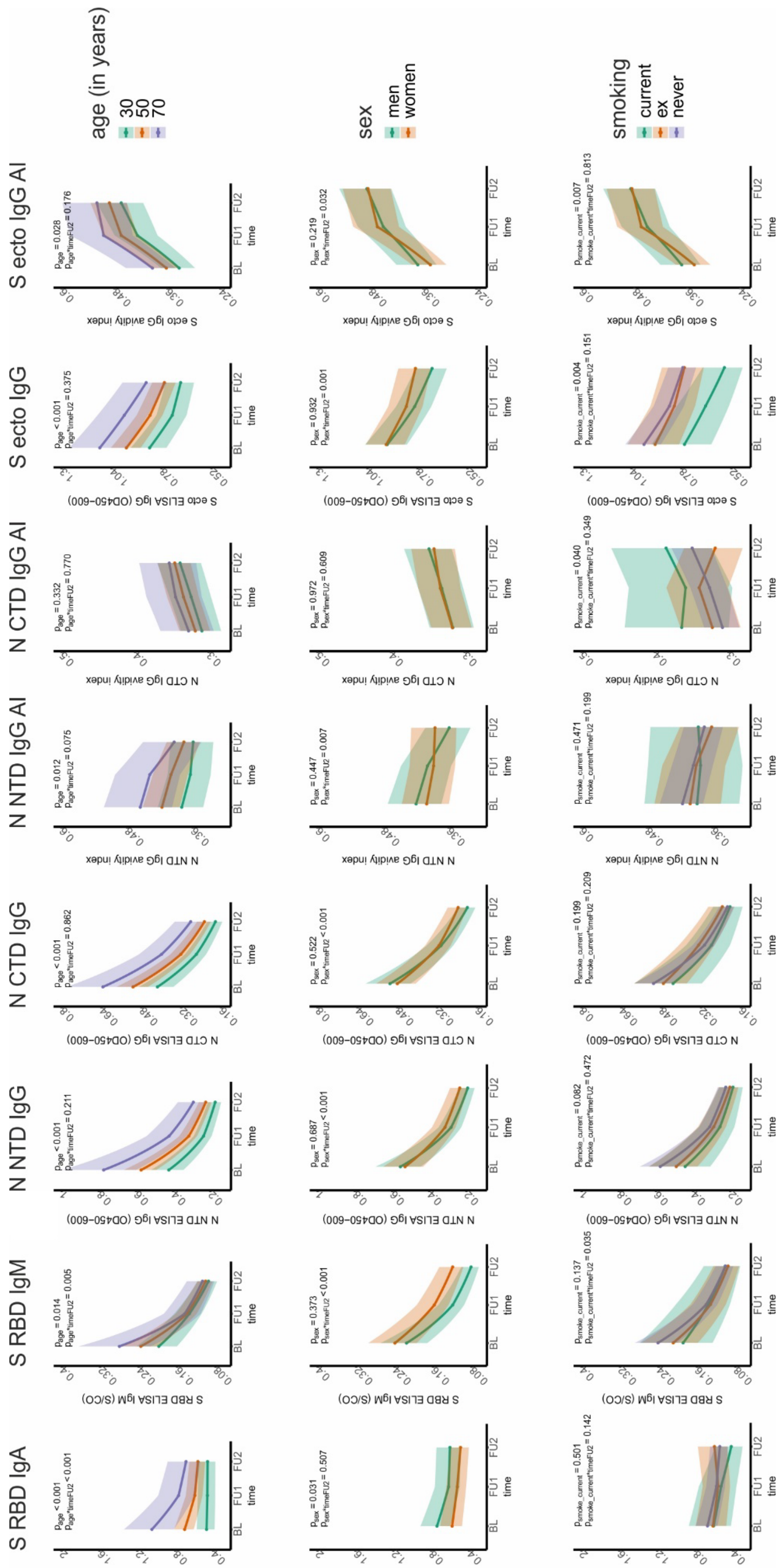

Supplementary Tables

Supplementary Table 1: Descriptive Statistics and sample sizes for all 13 measurements at time points BL, FU1 and FU2. Sample sizes are reported in square brackets. Standard deviations (SD) and Interquartile Range (IQR) are given in parentheses for mean and median, respectively.

	BL	FU1	FU2
**S RBD ELISA IgG (S/CO)**	**[n=211]**	**[n=211]**	**[n=211]**
Mean (SD)	3.54 (1.96)	2.64 (1.74)	2.29 (1.63)
Median (IQR)	3.12 (2.06-4.71)	2.11 (1.28-3.76)	1.85 (0.96-3.25)
Min, max	0.57, 9.39	0.30, 7.80	0.15, 8.51
**S RBD ELISA IgA (S/CO)**	**[n=211]**	**[n=211]**	**[n=211]**
Mean (SD)	1.03 (1.75)	0.85 (1.27)	0.85 (1.53)
Median (IQR)	0.59 (0.38-1.11)	0.56 (0.31-0.89)	0.5 (0.32-0.92)
Min, max	0.05, 21.43	0.07, 15.2	0.04, 17.95
**S RBD ELISA IgM (S/CO)**	**[n=211]**	**[n=211]**	**[n=211]**
Mean (SD)	0.37 (0.47)	0.2 (0.21)	0.15 (0.19)
Median (IQR)	0.22 (0.11-0.39)	0.13 (0.07-0.23)	0.09 (0.05-0.17)
Min, max	0.01, 3.79	0.02, 1.89	0.01, 1.93
**S Elecsys (COI)**	**[n=210]**	**[n=209]**	**[n=211]**
Mean (SD)	304.3 (461.7)	423.1 (701.5)	750 (4516.5)
Median (IQR)	128 (50.9-324.5)	169 (67.7-452)	171 (61.8-512.5)
Min, max	0.8, 2581	0.9, 5114	0.7, 65163
**N Elecsys (COI)**	**[n=211]**	**[n=211]**	**[n=211]**
Mean (SD)	75.4 (49.6)	63.5 (62.5)	41.4 (51.7)
Median (IQR)	79.4 (26.6-121.5)	43.2 (9.5-107)	22 (4.1-54.2)
Min, max	1.1, 155	0.5, 210	0.2, 251
**pseudovirus neutralization (ID50)**	**[n=210]**	**[n=209]**	**[n=207]**
Mean (SD)	168.5 (181.6)	132 (153.3)	144.3 (234.3)
Median (IQR)	120.2 (68.8-189.8)	98.2 (47-157.5)	79 (41.8-151.3)
Min, max	1, 1329	1, 1187	1, 2330
**N NTD ELISA IgG (OD450-600)**	**[n=191]**	**[n=210]**	**[n=211]**
Mean (SD)	0.72 (0.46)	0.43 (0.37)	0.32 (0.31)
Median (IQR)	0.64 (0.33-1.07)	0.29 (0.16-0.57)	0.2 (0.12-0.37)
Min, max	0.07, 1.7	0.04, 1.63	0.02, 1.57
**N CTD ELISA IgG (OD450-600)**	**[n=191]**	**[n=210]**	**[n=211]**
Mean (SD)	0.62 (0.36)	0.39 (0.28)	0.27 (0.2)
Median (IQR)	0.54 (0.32-0.9)	0.31 (0.19-0.52)	0.22 (0.14-0.35)
Min, max	0.04, 1.57	0.06, 1.46	0.04, 1.23

**N NTD IgG avidity index**	**[n=200]**	**[n=210]**	**[n=211]**
Mean (SD)	0.44 (0.17)	0.43 (0.16)	0.41 (0.16)
Median (IQR)	0.44 (0.32-0.53)	0.42 (0.32-0.53)	0.4 (0.29-0.52)
Min, max	0.07, 0.95	0.07, 0.86	0.06, 0.85
**N CTD IgG avidity index**	**[n=200]**	**[n=210]**	**[n=211]**
Mean (SD)	0.34 (0.15)	0.35 (0.11)	0.36 (0.11)
Median (IQR)	0.31 (0.25-0.41)	0.34 (0.27-0.42)	0.36 (0.29-0.43)
Min, max	0.1, 1.19	0.1, 0.74	0.05, 0.68
**S RBD IgG avidity index**	**[n=193]**	**[n=210]**	**[n=211]**
Mean (SD)	0.31 (0.14)	0.41 (0.17)	0.46 (0.18)
Median (IQR)	0.29 (0.21-0.38)	0.4 (0.28-0.51)	0.45 (0.32-0.57)
Min, max	0.07, 0.8	0.06, 0.96	0.06, 1.06
**S ecto ELISA IgG (OD450-600)**	**[n=188]**	**[n=207]**	**[n=208]**
Mean (SD)	1.02 (0.34)	0.89 (0.35)	0.84 (0.36)
Median (IQR)	1.01 (0.76-1.27)	0.88 (0.64-1.18)	0.85 (0.55-1.12)
Min, max	0.21, 1.69	0.25, 1.6	0.16, 1.71
**S ecto IgG avidity index**	**[n=186]**	**[n=207]**	**[n=208]**
Mean (SD)	0.41 (0.19)	0.5 (0.2)	0.53 (0.2)
Median (IQR)	0.38 (0.26-0.53)	0.48 (0.35-0.64)	0.52 (0.37-0.65)
Min, max	0.06, 0.87	0.05, 0.97	0.05, 1.04

Supplementary Table 2: Spearman's rho and P-values for correlation of the different serological parameters at the three visits. Spearman's rho values (green background) and P-values (blue background) corresponding to **Figure 2**.

Spearmans rho
p-value

		S Elecsys Ig	N Elecsys Ig	S RBD IgG	S RBD IgA	S RBD IgM	S ecto IgG	N NTD IgG	N CTD IgG	Neutralization	S RBD IgG AI	N NTD IgG AI	N CTD IgG AI	S ecto IgG AI
BL	S Elecsys Ig	1.0000	0.4865	0.7862	0.4534	0.1233	0.8224	0.5438	0.5366	0.6008	0.8334	0.3440	0.1855	0.8415
	N Elecsys Ig	<0.0001	1.0000	0.4721	0.2453	0.1522	0.4612	0.5168	0.8138	0.3844	0.3650	0.3711	0.2243	0.4036
	S RBD IgG	<0.0001	<0.0001	1.0000	0.5388	0.3004	0.8282	0.6372	0.5543	0.6913	0.6008	0.2975	0.1115	0.6038
	S RBD IgA	<0.0001	0.0003	<0.0001	1.0000	0.3010	0.4758	0.5165	0.4154	0.4703	0.3730	0.2219	0.1180	0.4371
	S RBD IgM	0.0747	0.0271	<0.0001	<0.0001	1.0000	0.2813	0.2379	0.1680	0.4086	0.0206	0.1009	0.0291	0.0368
	S ecto IgG	<0.0001	<0.0001	<0.0001	<0.0001	<0.0001	1.0000	0.6184	0.5988	0.6579	0.6432	0.3559	0.2365	0.7309
	N NTD IgG	<0.0001	<0.0001	<0.0001	<0.0001	0.0009	<0.0001	1.0000	0.6221	0.4802	0.4351	0.3928	0.1454	0.4486
	N CTD IgG	<0.0001	<0.0001	<0.0001	<0.0001	0.0202	<0.0001	<0.0001	1.0000	0.4077	0.4090	0.5069	0.3486	0.4783
	Neutralization	<0.0001	<0.0001	<0.0001	<0.0001	<0.0001	<0.0001	<0.0001	<0.0001	1.0000	0.5206	0.2213	0.1285	0.4613
	S RBD IgG AI	<0.0001	<0.0001	<0.0001	<0.0001	0.7761	<0.0001	<0.0001	<0.0001	<0.0001	1.0000	0.3468	0.2091	0.7752
	N NTD IgG AI	<0.0001	<0.0001	<0.0001	0.0016	0.1552	<0.0001	<0.0001	<0.0001	0.0017	<0.0001	1.0000	0.3765	0.3437
	N CTD IgG AI	0.0087	0.0014	0.1161	0.0960	0.6825	0.0011	0.0448	<0.0001	0.0706	0.0037	<0.0001	1.0000	0.2175
	S ecto IgG AI	<0.0001	<0.0001	<0.0001	<0.0001	0.6144	<0.0001	<0.0001	<0.0001	<0.0001	<0.0001	<0.0001	0.0027	1.0000
FU1	S Elecsys Ig	1.0000	0.5402	0.8122	0.5264	0.0029	0.8559	0.4317	0.4749	0.6202	0.8162	0.2190	-0.0632	0.8597
	N Elecsys Ig	<0.0001	1.0000	0.5217	0.3396	0.0681	0.5774	0.5878	0.8627	0.3900	0.4099	0.3741	-0.0577	0.4977
	S RBD IgG	<0.0001	<0.0001	1.0000	0.5064	0.1172	0.8805	0.4627	0.4823	0.5698	0.5035	0.2012	-0.0579	0.6432
	S RBD IgA	<0.0001	<0.0001	<0.0001	1.0000	0.1260	0.5630	0.3032	0.3435	0.3583	0.4245	0.2542	0.0084	0.4810
	S RBD IgM	0.9668	0.3251	0.0895	0.0677	1.0000	0.0812	0.1229	0.0753	0.1511	-0.0815	-0.0412	0.0207	-0.0387
	S ecto IgG	<0.0001	<0.0001	<0.0001	<0.0001	0.2449	1.0000	0.5017	0.5414	0.6582	0.6113	0.2420	-0.0383	0.7527
	N NTD IgG	<0.0001	<0.0001	<0.0001	<0.0001	0.0757	<0.0001	1.0000	0.5726	0.3524	0.3780	0.1591	0.0334	0.4022
	N CTD IgG	<0.0001	<0.0001	<0.0001	<0.0001	0.2772	<0.0001	<0.0001	1.0000	0.3019	0.3493	0.3236	0.0011	0.4271
	Neutralization	<0.0001	<0.0001	<0.0001	<0.0001	0.0290	<0.0001	<0.0001	<0.0001	1.0000	0.4777	0.1239	0.0404	0.5581
	S RBD IgG AI	<0.0001	<0.0001	<0.0001	<0.0001	0.2394	<0.0001	<0.0001	<0.0001	<0.0001	1.0000	0.2599	0.0156	0.8352
	N NTD IgG AI	0.0015	<0.0001	0.0034	0.0002	0.5527	0.0004	0.0211	<0.0001	0.0746	0.0001	1.0000	0.1378	0.2311
	N CTD IgG AI	0.3648	0.4057	0.4035	0.9036	0.7652	0.5833	0.6305	0.9871	0.5627	0.8224	0.0461	1.0000	0.0111
	S ecto IgG AI	<0.0001	<0.0001	<0.0001	<0.0001	0.5799	<0.0001	<0.0001	<0.0001	<0.0001	<0.0001	0.0008	0.8737	1.0000
FU2	S Elecsys Ig	1.0000	0.5498	0.8418	0.5652	0.0186	0.8568	0.3834	0.4546	0.5149	0.8123	0.1119	-0.1603	0.8686
	N Elecsys Ig	<0.0001	1.0000	0.5464	0.3277	0.0923	0.5855	0.5753	0.7857	0.4009	0.3456	0.3348	-0.1859	0.4652
	S RBD IgG	<0.0001	<0.0001	1.0000	0.5198	0.1144	0.8920	0.4288	0.4865	0.5813	0.5451	0.1246	-0.1235	0.6940
	S RBD IgA	<0.0001	<0.0001	<0.0001	1.0000	0.1172	0.5436	0.2533	0.3171	0.4168	0.4666	0.1483	-0.0518	0.5089
	S RBD IgM	0.7884	0.1816	0.0974	0.0894	1.0000	0.1142	0.1527	0.1294	0.1949	-0.0803	0.0183	-0.0618	-0.0187
	S ecto IgG	<0.0001	<0.0001	<0.0001	<0.0001	0.1006	1.0000	0.4377	0.5376	0.6130	0.5913	0.1027	-0.1367	0.7647
	N NTD IgG	<0.0001	<0.0001	<0.0001	0.0002	0.0266	<0.0001	1.0000	0.5470	0.2870	0.2066	-0.0341	-0.0712	0.3084
	N CTD IgG	<0.0001	<0.0001	<0.0001	<0.0001	0.0606	<0.0001	<0.0001	1.0000	0.2924	0.2530	0.2692	-0.1330	0.3923
	Neutralization	<0.0001	<0.0001	<0.0001	<0.0001	0.0046	<0.0001	<0.0001	<0.0001	1.0000	0.3559	0.0296	-0.0897	0.4687
	S RBD IgG AI	<0.0001	<0.0001	<0.0001	<0.0001	0.2456	<0.0001	0.0026	0.0002	<0.0001	1.0000	0.1898	-0.0706	0.8078
	N NTD IgG AI	0.1052	<0.0001	0.0710	0.0313	0.7921	0.1399	0.6221	0.0001	0.6700	0.0057	1.0000	0.2023	0.1167
	N CTD IgG AI	0.0198	0.0068	0.0735	0.4542	0.3714	0.0490	0.3036	0.0537	0.1953	0.3077	0.0032	1.0000	-0.0966
	S ecto IgG AI	<0.0001	<0.0001	<0.0001	<0.0001	0.7881	<0.0001	<0.0001	<0.0001	<0.0001	<0.0001	0.0931	0.1652	1.0000

Supplementary Table 3: Association of age, sex, and smoking on eight antibody and avidity levels and level change from linear mixed models. This table shows results from eight linear mixed models (LMMs), one for each of the following eight antibody and avidity levels as outcome: S RBD ELISA IgA, S RBD ELISA IgM, N NTD ELISA IgG, N CTD ELISA IgG, N NTD IgG AI, N CTD IgG AI, S ecto ELISA IgG, and S ecto ELISA IgG AI (ln-transformed to ensure approximate normal distribution). The covariates were 2 time-variables (timeFU1, timeFU2) and age (centered at 50 years), sex (1=women, 0=men), and 2 smoking variables (current smoking, ex-smoking versus never smokers as reference) as well as their interactions with the time-variables. For each of age, sex, and smoking variables, we show the covariate estimates and standard errors (s.e.) on the natural logarithm of levels (1st column) and on titer change between BL and FU1 or FU2 (2nd column or 3rd column, respectively; i.e. from the interaction terms with timeFU1 or timeFU2). In the first row per outcome, we show the intercept (1st column) and the association of timeFU1 or timeFU2 (2nd or 3rd column, respectively). The analogous results for the other 5 levels are shown in Table 3.

	Association with level coefficient (s.e.); P	Association with change until FU1	Association with change until FU2
**S RBD IgA**			
intercept/time point	-0.195 (s.e.: 0.092; p: 0.034)	-0.226 (s.e.: 0.049; p<0.001)	-0.275 (s.e.: 0.05; p<0.001)
age	0.020 (s.e.: 0.003; p<0.001)	-0.007 (s.e.: 0.002; p<0.001)	-0.010 (s.e.: 0.002; p<0.001)
sex	-0.244 (s.e.: 0.112; p: 0.031)	0.084 (s.e.: 0.059; p: 0.156)	0.039 (s.e.: 0.059; p: 0.507)
smoker_current	-0.102 (s.e.: 0.151; p: 0.501)	0.012 (s.e.: 0.107; p: 0.908)	-0.152 (s.e.: 0.103; p: 0.142)
smoker_ex	-0.09 (s.e.: 0.117; p: 0.441)	0.059 (s.e.: 0.072; p: 0.413)	0.189 (s.e.: 0.072; p: 0.009)
**S RBD IgM**			
intercept/time point	-1.446 (s.e.: 0.097; p<0.001)	-0.655 (s.e.: 0.055; p<0.001)	-1.043 (s.e.: 0.056; p<0.001)
age	0.009 (s.e.: 0.003; p: 0.014)	-0.007 (s.e.: 0.002; p<0.001)	-0.006 (s.e.: 0.002; p: 0.005)
sex	0.106 (s.e.: 0.119; p: 0.373)	0.172 (s.e.: 0.066; p: 0.01)	0.281 (s.e.: 0.067; p<0.001)
smoker_current	-0.245 (s.e.: 0.164; p: 0.137)	0.213 (s.e.: 0.12; p: 0.077)	0.247 (s.e.: 0.116; p: 0.035)
smoker_ex	-0.141 (s.e.: 0.126; p: 0.262)	0.075 (s.e.: 0.08; p: 0.353)	0.074 (s.e.: 0.08; p: 0.355)
**N NTD IgG**			
intercept/time point	-0.469 (s.e.: 0.094; p<0.001)	-0.707 (s.e.: 0.05; p<0.001)	-1.085 (s.e.: 0.05; p<0.001)
age	0.015 (s.e.: 0.003; p<0.001)	-0.001 (s.e.: 0.002; p: 0.715)	-0.002 (s.e.: 0.002; p: 0.211)
sex	-0.046 (s.e.: 0.115; p: 0.687)	0.157 (s.e.: 0.059; p: 0.008)	0.246 (s.e.: 0.059; p<0.001)
smoker_current	-0.264 (s.e.: 0.151; p: 0.082)	0.07 (s.e.: 0.107; p: 0.515)	0.075 (s.e.: 0.104; p: 0.472)
smoker_ex	-0.161 (s.e.: 0.118; p: 0.172)	0.091 (s.e.: 0.072; p: 0.209)	0.059 (s.e.: 0.073; p: 0.422)
**N CTD IgG**			
intercept/time point	-0.6 (s.e.: 0.077; p<0.001)	-0.558 (s.e.: 0.045; p<0.001)	-0.992 (s.e.: 0.045; p<0.001)
age	0.011 (s.e.: 0.003; p<0.001)	0.000 (s.e.: 0.002; p: 0.906)	0.000 (s.e.: 0.002; p: 0.862)
sex	-0.06 (s.e.: 0.094; p: 0.522)	0.088 (s.e.: 0.053; p: 0.101)	0.231 (s.e.: 0.054; p<0.001)
smoker_current	-0.167 (s.e.: 0.129; p: 0.199)	0.071 (s.e.: 0.097; p: 0.466)	0.118 (s.e.: 0.094; p: 0.209)
smoker_ex	-0.081 (s.e.: 0.099; p: 0.417)	0.155 (s.e.: 0.065; p: 0.018)	0.172 (s.e.: 0.066; p: 0.01)
**N NTD IgG AI**			
intercept/time point	-0.836 (s.e.: 0.051; p<0.001)	-0.059 (s.e.: 0.036; p: 0.104)	-0.173 (s.e.: 0.036; p<0.001)
age	0.005 (s.e.: 0.002; p: 0.012)	0.000 (s.e.: 0.001; p: 0.944)	-0.002 (s.e.: 0.001; p: 0.075)
sex	-0.047 (s.e.: 0.062; p: 0.447)	0.018 (s.e.: 0.043; p: 0.668)	0.117 (s.e.: 0.043; p: 0.007)
smoker_current	-0.067 (s.e.: 0.093; p: 0.471)	0.033 (s.e.: 0.077; p: 0.67)	0.096 (s.e.: 0.075; p: 0.199)
smoker_ex	-0.035 (s.e.: 0.068; p: 0.614)	0.021 (s.e.: 0.051; p: 0.678)	-0.002 (s.e.: 0.052; p: 0.971)

**N CTD IgG AI**			
intercept/time point	-1.173 (s.e.: 0.042; p<0.001)	0.054 (s.e.: 0.036; p: 0.13)	0.136 (s.e.: 0.036; p<0.001)
age	0.001 (s.e.: 0.001; p: 0.332)	0.000 (s.e.: 0.001; p: 0.897)	0.000 (s.e.: 0.001; p: 0.77)
sex	0.002 (s.e.: 0.05; p: 0.972)	0.004 (s.e.: 0.042; p: 0.926)	-0.022 (s.e.: 0.042; p: 0.609)
smoker_current	0.165 (s.e.: 0.08; p: 0.04)	-0.07 (s.e.: 0.076; p: 0.357)	-0.069 (s.e.: 0.073; p: 0.349)
smoker_ex	0.044 (s.e.: 0.058; p: 0.448)	-0.001 (s.e.: 0.051; p: 0.981)	-0.138 (s.e.: 0.051; p: 0.008)
**S ecto IgG**			
intercept/time point	-0.005 (s.e.: 0.05; p: 0.926)	-0.17 (s.e.: 0.027; p<0.001)	-0.294 (s.e.: 0.028; p<0.001)
age	0.007 (s.e.: 0.002; p<0.001)	0.001 (s.e.: 0.001; p: 0.473)	-0.001 (s.e.: 0.001; p: 0.375)
sex	0.005 (s.e.: 0.062; p: 0.932)	0.048 (s.e.: 0.033; p: 0.143)	0.111 (s.e.: 0.033; p: 0.001)
smoker_current	-0.243 (s.e.: 0.085; p: 0.004)	-0.009 (s.e.: 0.062; p: 0.885)	-0.085 (s.e.: 0.059; p: 0.151)
smoker_ex	-0.061 (s.e.: 0.066; p: 0.354)	0.031 (s.e.: 0.04; p: 0.444)	0.051 (s.e.: 0.041; p: 0.212)
**S ecto IgG AI**			
intercept/time point	-0.945 (s.e.: 0.053; p<0.001)	0.189 (s.e.: 0.029; p<0.001)	0.25 (s.e.: 0.029; p<0.001)
age	0.004 (s.e.: 0.002; p: 0.028)	0.000 (s.e.: 0.001; p: 0.952)	-0.001 (s.e.: 0.001; p: 0.176)
sex	-0.079 (s.e.: 0.064; p: 0.219)	0.109 (s.e.: 0.034; p: 0.002)	0.074 (s.e.: 0.034; p: 0.032)
smoker_current	-0.237 (s.e.: 0.088; p: 0.007)	-0.033 (s.e.: 0.064; p: 0.608)	0.015 (s.e.: 0.061; p: 0.813)
smoker_ex	-0.028 (s.e.: 0.069; p: 0.688)	0.018 (s.e.: 0.042; p: 0.664)	0.057 (s.e.: 0.042; p: 0.176)

Supplementary Table 4: Linear Mixed Model regression output for 13 assays. Linear Mixed Model (LMM) regression output for each of the 13 assays (outcome, log-transformed). The model in the column "smoking" is the main model (including the covariates timeFU1, timeFU2 (two time variables), age (years), sex (1=women, 0=men), and 2 smoking variables (current smoker, ex-smoker, never smoker as reference) as well as their interaction with timeFU1 and timeFU2; the model in the column "base" is the same, except it does not include the 2 smoking variables; and the model in the column "symptomatic" additionally includes symptoms (yes or maybe versus asymptomatic as reference) and their interaction with age. Beta estimates, standard errors (s.e.) and P-values are reported.

outcome	beta estimates	base	smoking	symptomatic
log(S RBD IgG)	intercept	1.195 (s.e.: 0.068; p<0.001)	1.265 (s.e.: 0.075; p<0.001)	1.159 (s.e.: 0.134; p<0.001)
	timeFU1	-0.423 (s.e.: 0.035; p<0.001)	-0.462 (s.e.: 0.04; p<0.001)	-0.461 (s.e.: 0.04; p<0.001)
	timeFU2	-0.638 (s.e.: 0.035; p<0.001)	-0.658 (s.e.: 0.04; p<0.001)	-0.658 (s.e.: 0.04; p<0.001)
	age	0.012 (s.e.: 0.003; p<0.001)	0.013 (s.e.: 0.003; p<0.001)	0.006 (s.e.: 0.006; p: 0.377)
	sex	-0.091 (s.e.: 0.093; p: 0.329)	-0.104 (s.e.: 0.091; p: 0.258)	-0.076 (s.e.: 0.091; p: 0.402)
	smoker_current	n.a.	-0.402 (s.e.: 0.122; p: 0.001)	-0.409 (s.e.: 0.122; p: 0.001)
	smoker_ex	n.a.	-0.111 (s.e.: 0.094; p: 0.239)	-0.12 (s.e.: 0.095; p: 0.206)
	symptomatic_yes	n.a.	n.a.	0.236 (s.e.: 0.149; p: 0.115)
	symptomatic_maybe	n.a.	n.a.	0.053 (s.e.: 0.129; p: 0.679)
	age*timeFU1	0.001 (s.e.: 0.001; p: 0.3)	0.001 (s.e.: 0.001; p: 0.359)	0.001 (s.e.: 0.001; p: 0.357)
	age*timeFU2	0 (s.e.: 0.001; p: 0.787)	-0.001 (s.e.: 0.001; p: 0.464)	-0.001 (s.e.: 0.001; p: 0.469)
	sex*timeFU1	0.122 (s.e.: 0.048; p: 0.012)	0.13 (s.e.: 0.047; p: 0.006)	0.13 (s.e.: 0.047; p: 0.006)
	sex*timeFU2	0.18 (s.e.: 0.048; p<0.001)	0.166 (s.e.: 0.048; p: 0.001)	0.166 (s.e.: 0.048; p: 0.001)
	smoker_current*timeFU1	n.a.	0.054 (s.e.: 0.086; p: 0.527)	0.055 (s.e.: 0.086; p: 0.524)
	smoker_current*timeFU2	n.a.	-0.027 (s.e.: 0.083; p: 0.744)	-0.026 (s.e.: 0.083; p: 0.753)
	smoker_ex*timeFU1	n.a.	0.112 (s.e.: 0.057; p: 0.051)	0.112 (s.e.: 0.057; p: 0.052)
	smoker_ex*timeFU2	n.a.	0.135 (s.e.: 0.058; p: 0.021)	0.134 (s.e.: 0.058; p: 0.022)
	age*symptomatic_yes	n.a.	n.a.	0.018 (s.e.: 0.008; p: 0.027)
	age*symptomatic_maybe	n.a.	n.a.	0.004 (s.e.: 0.007; p: 0.567)
log(S RBD IgA)	intercept	-0.229 (s.e.: 0.083; p: 0.006)	-0.195 (s.e.: 0.092; p: 0.034)	-0.471 (s.e.: 0.165; p: 0.005)
	timeFU1	-0.208 (s.e.: 0.044; p<0.001)	-0.226 (s.e.: 0.049; p<0.001)	-0.226 (s.e.: 0.049; p<0.001)
	timeFU2	-0.248 (s.e.: 0.044; p<0.001)	-0.275 (s.e.: 0.05; p<0.001)	-0.275 (s.e.: 0.05; p<0.001)
	age	0.02 (s.e.: 0.003; p<0.001)	0.02 (s.e.: 0.003; p<0.001)	0.007 (s.e.: 0.008; p: 0.371)
	sex	-0.239 (s.e.: 0.113; p: 0.034)	-0.244 (s.e.: 0.112; p: 0.031)	-0.218 (s.e.: 0.112; p: 0.053)
	smoker_current	n.a.	-0.102 (s.e.: 0.151; p: 0.501)	-0.091 (s.e.: 0.151; p: 0.546)
	smoker_ex	n.a.	-0.09 (s.e.: 0.117; p: 0.441)	-0.08 (s.e.: 0.117; p: 0.496)
	symptomatic_yes	n.a.	n.a.	0.371 (s.e.: 0.184; p: 0.044)
	symptomatic_maybe	n.a.	n.a.	0.277 (s.e.: 0.159; p: 0.082)
	age*timeFU1	-0.007 (s.e.: 0.002; p<0.001)	-0.007 (s.e.: 0.002; p<0.001)	-0.007 (s.e.: 0.002; p<0.001)
	age*timeFU2	-0.009 (s.e.: 0.002; p<0.001)	-0.01 (s.e.: 0.002; p<0.001)	-0.01 (s.e.: 0.002; p<0.001)
	sex*timeFU1	0.081 (s.e.: 0.059; p: 0.172)	0.084 (s.e.: 0.059; p: 0.156)	0.084 (s.e.: 0.059; p: 0.156)
	sex*timeFU2	0.046 (s.e.: 0.059; p: 0.442)	0.039 (s.e.: 0.059; p: 0.507)	0.039 (s.e.: 0.059; p: 0.507)
	smoker_current*timeFU1	n.a.	0.012 (s.e.: 0.107; p: 0.908)	0.013 (s.e.: 0.107; p: 0.906)
	smoker_current*timeFU2	n.a.	-0.152 (s.e.: 0.103; p: 0.142)	-0.151 (s.e.: 0.103; p: 0.146)

outcome	beta estimates	base	smoking	symptomatic
	smoker_ex*timeFU1	n.a.	0.059 (s.e.: 0.072; p: 0.413)	0.058 (s.e.: 0.072; p: 0.417)
	smoker_ex*timeFU2	n.a.	0.189 (s.e.: 0.072; p: 0.009)	0.188 (s.e.: 0.072; p: 0.01)
	age*symptomatic_yes	n.a.	n.a.	0.021 (s.e.: 0.01; p: 0.043)
	age*symptomatic_maybe	n.a.	n.a.	0.013 (s.e.: 0.009; p: 0.126)
log(S RBD IgM)	intercept	-1.508 (s.e.: 0.088; p<0.001)	-1.446 (s.e.: 0.097; p<0.001)	-1.643 (s.e.: 0.174; p<0.001)
	timeFU1	-0.614 (s.e.: 0.049; p<0.001)	-0.655 (s.e.: 0.055; p<0.001)	-0.655 (s.e.: 0.055; p<0.001)
	timeFU2	-0.996 (s.e.: 0.049; p<0.001)	-1.043 (s.e.: 0.056; p<0.001)	-1.043 (s.e.: 0.056; p<0.001)
	age	0.008 (s.e.: 0.003; p: 0.017)	0.009 (s.e.: 0.003; p: 0.014)	-0.01 (s.e.: 0.008; p: 0.223)
	sex	0.116 (s.e.: 0.119; p: 0.332)	0.106 (s.e.: 0.119; p: 0.373)	0.124 (s.e.: 0.119; p: 0.297)
	smoker_current	n.a.	-0.245 (s.e.: 0.164; p: 0.137)	-0.246 (s.e.: 0.165; p: 0.136)
	smoker_ex	n.a.	-0.141 (s.e.: 0.126; p: 0.262)	-0.137 (s.e.: 0.126; p: 0.279)
	symptomatic_yes	n.a.	n.a.	0.126 (s.e.: 0.194; p: 0.514)
	symptomatic_maybe	n.a.	n.a.	0.262 (s.e.: 0.167; p: 0.117)
	age*timeFU1	-0.007 (s.e.: 0.002; p<0.001)	-0.007 (s.e.: 0.002; p<0.001)	-0.007 (s.e.: 0.002; p<0.001)
	age*timeFU2	-0.006 (s.e.: 0.002; p: 0.004)	-0.006 (s.e.: 0.002; p: 0.005)	-0.006 (s.e.: 0.002; p: 0.005)
	sex*timeFU1	0.164 (s.e.: 0.066; p: 0.013)	0.172 (s.e.: 0.066; p: 0.01)	0.172 (s.e.: 0.066; p: 0.01)
	sex*timeFU2	0.27 (s.e.: 0.066; p<0.001)	0.281 (s.e.: 0.067; p<0.001)	0.281 (s.e.: 0.067; p<0.001)
	smoker_current*timeFU1	n.a.	0.213 (s.e.: 0.12; p: 0.077)	0.213 (s.e.: 0.12; p: 0.077)
	smoker_current*timeFU2	n.a.	0.247 (s.e.: 0.116; p: 0.035)	0.249 (s.e.: 0.116; p: 0.033)
	smoker_ex*timeFU1	n.a.	0.075 (s.e.: 0.08; p: 0.353)	0.074 (s.e.: 0.08; p: 0.355)
	smoker_ex*timeFU2	n.a.	0.084 (s.e.: 0.082; p: 0.302)	0.084 (s.e.: 0.082; p: 0.305)
	age*symptomatic_yes	n.a.	n.a.	0.02 (s.e.: 0.011; p: 0.062)
	age*symptomatic_maybe	n.a.	n.a.	0.024 (s.e.: 0.009; p: 0.011)
log(S Elecsys Ig)	intercept	5.077 (s.e.: 0.145; p<0.001)	5.186 (s.e.: 0.156; p<0.001)	5.053 (s.e.: 0.28; p<0.001)
	timeFU1	0.124 (s.e.: 0.062; p: 0.047)	0.073 (s.e.: 0.069; p: 0.291)	0.074 (s.e.: 0.069; p: 0.289)
	timeFU2	0.123 (s.e.: 0.062; p: 0.049)	0.078 (s.e.: 0.07; p: 0.262)	0.079 (s.e.: 0.07; p: 0.258)
	age	0.021 (s.e.: 0.006; p<0.001)	0.021 (s.e.: 0.006; p<0.001)	0.022 (s.e.: 0.013; p: 0.102)
	sex	-0.263 (s.e.: 0.198; p: 0.183)	-0.283 (s.e.: 0.194; p: 0.144)	-0.221 (s.e.: 0.189; p: 0.244)
	smoker_current	n.a.	-0.628 (s.e.: 0.236; p: 0.008)	-0.661 (s.e.: 0.235; p: 0.005)
	smoker_ex	n.a.	-0.176 (s.e.: 0.188; p: 0.349)	-0.205 (s.e.: 0.186; p: 0.271)
	symptomatic_yes	n.a.	n.a.	0.45 (s.e.: 0.315; p: 0.154)
	symptomatic_maybe	n.a.	n.a.	-0.038 (s.e.: 0.272; p: 0.888)
	age*timeFU1	-0.001 (s.e.: 0.002; p: 0.834)	-0.001 (s.e.: 0.002; p: 0.736)	-0.001 (s.e.: 0.002; p: 0.744)
	age*timeFU2	-0.001 (s.e.: 0.002; p: 0.6)	-0.003 (s.e.: 0.002; p: 0.234)	-0.003 (s.e.: 0.002; p: 0.239)
	sex*timeFU1	0.246 (s.e.: 0.085; p: 0.004)	0.253 (s.e.: 0.083; p: 0.003)	0.253 (s.e.: 0.083; p: 0.003)
	sex*timeFU2	0.31 (s.e.: 0.085; p<0.001)	0.279 (s.e.: 0.084; p: 0.001)	0.278 (s.e.: 0.084; p: 0.001)

outcome	beta estimates	base	smoking	symptomatic
	smoker_current*timeFU1	n.a.	-0.069 (s.e.: 0.151; p: 0.645)	-0.07 (s.e.: 0.151; p: 0.645)
	smoker_current*timeFU2	n.a.	-0.17 (s.e.: 0.146; p: 0.247)	-0.168 (s.e.: 0.146; p: 0.252)
	smoker_ex*timeFU1	n.a.	0.214 (s.e.: 0.101; p: 0.035)	0.213 (s.e.: 0.101; p: 0.036)
	smoker_ex*timeFU2	n.a.	0.324 (s.e.: 0.102; p: 0.002)	0.323 (s.e.: 0.102; p: 0.002)
	age*symptomatic_yes	n.a.	n.a.	0.034 (s.e.: 0.018; p: 0.058)
	age*symptomatic_maybe	n.a.	n.a.	-0.015 (s.e.: 0.015; p: 0.32)
log(N Elecsys Ig)	intercept	3.965 (s.e.: 0.145; p<0.001)	4.035 (s.e.: 0.158; p<0.001)	3.396 (s.e.: 0.28; p<0.001)
	timeFU1	-0.472 (s.e.: 0.071; p<0.001)	-0.448 (s.e.: 0.08; p<0.001)	-0.448 (s.e.: 0.08; p<0.001)
	timeFU2	-1.244 (s.e.: 0.071; p<0.001)	-1.149 (s.e.: 0.081; p<0.001)	-1.149 (s.e.: 0.081; p<0.001)
	age	0.011 (s.e.: 0.006; p: 0.058)	0.01 (s.e.: 0.006; p: 0.066)	0.008 (s.e.: 0.013; p: 0.556)
	sex	-0.085 (s.e.: 0.197; p: 0.665)	-0.1 (s.e.: 0.193; p: 0.604)	-0.044 (s.e.: 0.19; p: 0.817)
	smoker_current	n.a.	-0.528 (s.e.: 0.253; p: 0.038)	-0.509 (s.e.: 0.251; p: 0.044)
	smoker_ex	n.a.	-0.069 (s.e.: 0.197; p: 0.725)	-0.031 (s.e.: 0.196; p: 0.873)
	symptomatic_yes	n.a.	n.a.	0.808 (s.e.: 0.312; p: 0.01)
	symptomatic_maybe	n.a.	n.a.	0.617 (s.e.: 0.269; p: 0.022)
	age*timeFU1	0.014 (s.e.: 0.003; p<0.001)	0.015 (s.e.: 0.003; p<0.001)	0.015 (s.e.: 0.003; p<0.001)
	age*timeFU2	0.02 (s.e.: 0.003; p<0.001)	0.021 (s.e.: 0.003; p<0.001)	0.021 (s.e.: 0.003; p<0.001)
	sex*timeFU1	0.046 (s.e.: 0.097; p: 0.639)	0.049 (s.e.: 0.096; p: 0.613)	0.048 (s.e.: 0.096; p: 0.615)
	sex*timeFU2	0.31 (s.e.: 0.097; p: 0.001)	0.287 (s.e.: 0.097; p: 0.003)	0.286 (s.e.: 0.097; p: 0.003)
	smoker_current*timeFU1	n.a.	-0.179 (s.e.: 0.174; p: 0.305)	-0.18 (s.e.: 0.174; p: 0.302)
	smoker_current*timeFU2	n.a.	-0.424 (s.e.: 0.169; p: 0.012)	-0.42 (s.e.: 0.169; p: 0.013)
	smoker_ex*timeFU1	n.a.	-0.037 (s.e.: 0.117; p: 0.748)	-0.039 (s.e.: 0.117; p: 0.741)
	smoker_ex*timeFU2	n.a.	-0.094 (s.e.: 0.118; p: 0.426)	-0.096 (s.e.: 0.118; p: 0.415)
	age*symptomatic_yes	n.a.	n.a.	0.032 (s.e.: 0.017; p: 0.066)
	age*symptomatic_maybe	n.a.	n.a.	-0.007 (s.e.: 0.015; p: 0.635)
log(Neutralization)	intercept	4.832 (s.e.: 0.109; p<0.001)	4.943 (s.e.: 0.123; p<0.001)	4.593 (s.e.: 0.201; p<0.001)
	timeFU1	-0.388 (s.e.: 0.107; p<0.001)	-0.446 (s.e.: 0.121; p<0.001)	-0.446 (s.e.: 0.121; p<0.001)
	timeFU2	-0.621 (s.e.: 0.107; p<0.001)	-0.594 (s.e.: 0.123; p<0.001)	-0.593 (s.e.: 0.123; p<0.001)
	age	0.02 (s.e.: 0.004; p<0.001)	0.02 (s.e.: 0.004; p<0.001)	0.02 (s.e.: 0.009; p: 0.035)
	sex	-0.008 (s.e.: 0.149; p: 0.959)	-0.026 (s.e.: 0.148; p: 0.861)	0 (s.e.: 0.148; p: 1)
	smoker_current	n.a.	-0.55 (s.e.: 0.249; p: 0.028)	-0.529 (s.e.: 0.249; p: 0.034)
	smoker_ex	n.a.	-0.212 (s.e.: 0.176; p: 0.227)	-0.181 (s.e.: 0.176; p: 0.304)
	symptomatic_yes	n.a.	n.a.	0.414 (s.e.: 0.211; p: 0.05)
	symptomatic_maybe	n.a.	n.a.	0.351 (s.e.: 0.183; p: 0.055)
	age*timeFU1	-0.005 (s.e.: 0.004; p: 0.198)	-0.005 (s.e.: 0.004; p: 0.216)	-0.005 (s.e.: 0.004; p: 0.217)

outcome	beta estimates	base	smoking	symptomatic
	age*timeFU2	0.002 (s.e.: 0.004; p: 0.65)	0.002 (s.e.: 0.004; p: 0.617)	0.002 (s.e.: 0.004; p: 0.608)
	sex*timeFU1	0.024 (s.e.: 0.146; p: 0.872)	0.034 (s.e.: 0.146; p: 0.813)	0.034 (s.e.: 0.146; p: 0.817)
	sex*timeFU2	0.262 (s.e.: 0.146; p: 0.074)	0.243 (s.e.: 0.147; p: 0.1)	0.239 (s.e.: 0.147; p: 0.106)
	smoker_current*timeFU1	n.a.	0.286 (s.e.: 0.263; p: 0.277)	0.284 (s.e.: 0.263; p: 0.281)
	smoker_current*timeFU2	n.a.	-0.009 (s.e.: 0.258; p: 0.974)	-0.003 (s.e.: 0.258; p: 0.989)
	smoker_ex*timeFU1	n.a.	0.11 (s.e.: 0.177; p: 0.535)	0.108 (s.e.: 0.177; p: 0.543)
	smoker_ex*timeFU2	n.a.	-0.04 (s.e.: 0.179; p: 0.824)	-0.044 (s.e.: 0.179; p: 0.804)
	age*symptomatic_yes	n.a.	n.a.	0.014 (s.e.: 0.012; p: 0.236)
	age*symptomatic_maybe	n.a.	n.a.	-0.004 (s.e.: 0.01; p: 0.693)
log(N NTD IgG)	intercept	-0.54 (s.e.: 0.085; p<0.001)	-0.469 (s.e.: 0.094; p<0.001)	-0.767 (s.e.: 0.168; p<0.001)
	timeFU1	-0.674 (s.e.: 0.043; p<0.001)	-0.707 (s.e.: 0.05; p<0.001)	-0.706 (s.e.: 0.05; p<0.001)
	timeFU2	-1.066 (s.e.: 0.043; p<0.001)	-1.085 (s.e.: 0.05; p<0.001)	-1.085 (s.e.: 0.05; p<0.001)
	age	0.015 (s.e.: 0.003; p<0.001)	0.015 (s.e.: 0.003; p<0.001)	0.01 (s.e.: 0.008; p: 0.23)
	sex	-0.033 (s.e.: 0.115; p: 0.773)	-0.046 (s.e.: 0.115; p: 0.687)	-0.018 (s.e.: 0.115; p: 0.877)
	smoker_current	n.a.	-0.264 (s.e.: 0.151; p: 0.082)	-0.256 (s.e.: 0.152; p: 0.092)
	smoker_ex	n.a.	-0.161 (s.e.: 0.118; p: 0.172)	-0.148 (s.e.: 0.119; p: 0.214)
	symptomatic_yes	n.a.	n.a.	0.353 (s.e.: 0.187; p: 0.06)
	symptomatic_maybe	n.a.	n.a.	0.299 (s.e.: 0.162; p: 0.065)
	age*timeFU1	0 (s.e.: 0.002; p: 0.824)	-0.001 (s.e.: 0.002; p: 0.715)	-0.001 (s.e.: 0.002; p: 0.716)
	age*timeFU2	-0.002 (s.e.: 0.002; p: 0.244)	-0.002 (s.e.: 0.002; p: 0.211)	-0.002 (s.e.: 0.002; p: 0.215)
	sex*timeFU1	0.148 (s.e.: 0.058; p: 0.011)	0.157 (s.e.: 0.059; p: 0.008)	0.157 (s.e.: 0.059; p: 0.008)
	sex*timeFU2	0.242 (s.e.: 0.058; p<0.001)	0.246 (s.e.: 0.059; p<0.001)	0.246 (s.e.: 0.059; p<0.001)
	smoker_current*timeFU1	n.a.	0.07 (s.e.: 0.107; p: 0.515)	0.069 (s.e.: 0.107; p: 0.522)
	smoker_current*timeFU2	n.a.	0.075 (s.e.: 0.104; p: 0.472)	0.075 (s.e.: 0.104; p: 0.467)
	smoker_ex*timeFU1	n.a.	0.091 (s.e.: 0.072; p: 0.209)	0.09 (s.e.: 0.072; p: 0.214)
	smoker_ex*timeFU2	n.a.	0.059 (s.e.: 0.073; p: 0.422)	0.057 (s.e.: 0.073; p: 0.435)
	age*symptomatic_yes	n.a.	n.a.	0.019 (s.e.: 0.011; p: 0.077)
	age*symptomatic_maybe	n.a.	n.a.	0.002 (s.e.: 0.009; p: 0.792)
log(N CTD IgG)	intercept	-0.639 (s.e.: 0.069; p<0.001)	-0.6 (s.e.: 0.077; p<0.001)	-0.873 (s.e.: 0.135; p<0.001)
	timeFU1	-0.507 (s.e.: 0.039; p<0.001)	-0.558 (s.e.: 0.045; p<0.001)	-0.557 (s.e.: 0.045; p<0.001)
	timeFU2	-0.935 (s.e.: 0.039; p<0.001)	-0.992 (s.e.: 0.045; p<0.001)	-0.991 (s.e.: 0.045; p<0.001)
	age	0.01 (s.e.: 0.003; p<0.001)	0.011 (s.e.: 0.003; p<0.001)	0.003 (s.e.: 0.006; p: 0.623)
	sex	-0.05 (s.e.: 0.094; p: 0.592)	-0.06 (s.e.: 0.094; p: 0.522)	-0.034 (s.e.: 0.093; p: 0.712)
	smoker_current	n.a.	-0.167 (s.e.: 0.129; p: 0.199)	-0.157 (s.e.: 0.129; p: 0.225)
	smoker_ex	n.a.	-0.081 (s.e.: 0.099; p: 0.417)	-0.068 (s.e.: 0.099; p: 0.491)
	symptomatic_yes	n.a.	n.a.	0.424 (s.e.: 0.149; p: 0.005)

outcome	beta estimates	base	smoking	symptomatic
	symptomatic_maybe	n.a.	n.a.	0.244 (s.e.: 0.129; p: 0.059)
	age*timeFU1	0.001 (s.e.: 0.002; p: 0.585)	0 (s.e.: 0.002; p: 0.906)	0 (s.e.: 0.002; p: 0.905)
	age*timeFU2	0.001 (s.e.: 0.002; p: 0.461)	0 (s.e.: 0.002; p: 0.862)	0 (s.e.: 0.002; p: 0.854)
	sex*timeFU1	0.077 (s.e.: 0.053; p: 0.147)	0.088 (s.e.: 0.053; p: 0.101)	0.087 (s.e.: 0.053; p: 0.102)
	sex*timeFU2	0.222 (s.e.: 0.053; p<0.001)	0.231 (s.e.: 0.054; p<0.001)	0.231 (s.e.: 0.054; p<0.001)
	smoker_current*timeFU1	n.a.	0.071 (s.e.: 0.097; p: 0.466)	0.07 (s.e.: 0.097; p: 0.469)
	smoker_current*timeFU2	n.a.	0.118 (s.e.: 0.094; p: 0.209)	0.119 (s.e.: 0.094; p: 0.204)
	smoker_ex*timeFU1	n.a.	0.155 (s.e.: 0.065; p: 0.018)	0.154 (s.e.: 0.065; p: 0.019)
	smoker_ex*timeFU2	n.a.	0.172 (s.e.: 0.066; p: 0.01)	0.17 (s.e.: 0.066; p: 0.01)
	age*symptomatic_yes	n.a.	n.a.	0.017 (s.e.: 0.008; p: 0.038)
	age*symptomatic_maybe	n.a.	n.a.	0.005 (s.e.: 0.007; p: 0.444)
log(N NTD IgG AI)	intercept	-0.853 (s.e.: 0.045; p<0.001)	-0.836 (s.e.: 0.051; p<0.001)	-0.883 (s.e.: 0.089; p<0.001)
	timeFU1	-0.049 (s.e.: 0.032; p: 0.123)	-0.059 (s.e.: 0.036; p: 0.104)	-0.058 (s.e.: 0.036; p: 0.109)
	timeFU2	-0.162 (s.e.: 0.032; p<0.001)	-0.173 (s.e.: 0.036; p<0.001)	-0.172 (s.e.: 0.036; p<0.001)
	age	0.004 (s.e.: 0.002; p: 0.012)	0.005 (s.e.: 0.002; p: 0.012)	0.007 (s.e.: 0.004; p: 0.098)
	sex	-0.043 (s.e.: 0.061; p: 0.482)	-0.047 (s.e.: 0.062; p: 0.447)	-0.037 (s.e.: 0.062; p: 0.544)
	smoker_current	n.a.	-0.067 (s.e.: 0.093; p: 0.471)	-0.067 (s.e.: 0.093; p: 0.472)
	smoker_ex	n.a.	-0.035 (s.e.: 0.068; p: 0.614)	-0.034 (s.e.: 0.069; p: 0.617)
	symptomatic_yes	n.a.	n.a.	0.118 (s.e.: 0.097; p: 0.222)
	symptomatic_maybe	n.a.	n.a.	0.013 (s.e.: 0.083; p: 0.875)
	age*timeFU1	0 (s.e.: 0.001; p: 0.918)	0 (s.e.: 0.001; p: 0.944)	0 (s.e.: 0.001; p: 0.938)
	age*timeFU2	-0.002 (s.e.: 0.001; p: 0.05)	-0.002 (s.e.: 0.001; p: 0.075)	-0.002 (s.e.: 0.001; p: 0.078)
	sex*timeFU1	0.015 (s.e.: 0.042; p: 0.717)	0.018 (s.e.: 0.043; p: 0.668)	0.018 (s.e.: 0.043; p: 0.675)
	sex*timeFU2	0.108 (s.e.: 0.042; p: 0.011)	0.117 (s.e.: 0.043; p: 0.007)	0.116 (s.e.: 0.043; p: 0.007)
	smoker_current*timeFU1	n.a.	0.033 (s.e.: 0.077; p: 0.67)	0.032 (s.e.: 0.077; p: 0.674)
	smoker_current*timeFU2	n.a.	0.096 (s.e.: 0.075; p: 0.199)	0.096 (s.e.: 0.075; p: 0.198)
	smoker_ex*timeFU1	n.a.	0.021 (s.e.: 0.051; p: 0.678)	0.02 (s.e.: 0.051; p: 0.691)
	smoker_ex*timeFU2	n.a.	-0.002 (s.e.: 0.052; p: 0.971)	-0.003 (s.e.: 0.052; p: 0.955)
	age*symptomatic_yes	n.a.	n.a.	0.004 (s.e.: 0.005; p: 0.51)
	age*symptomatic_maybe	n.a.	n.a.	-0.005 (s.e.: 0.005; p: 0.248)
log(N CTD IgG AI)	intercept	-1.143 (s.e.: 0.037; p<0.001)	-1.173 (s.e.: 0.042; p<0.001)	-1.147 (s.e.: 0.071; p<0.001)
	timeFU1	0.045 (s.e.: 0.031; p: 0.148)	0.054 (s.e.: 0.036; p: 0.13)	0.054 (s.e.: 0.036; p: 0.131)
	timeFU2	0.095 (s.e.: 0.031; p: 0.003)	0.136 (s.e.: 0.036; p<0.001)	0.136 (s.e.: 0.036; p<0.001)
	age	0.001 (s.e.: 0.001; p: 0.319)	0.001 (s.e.: 0.001; p: 0.332)	0.001 (s.e.: 0.003; p: 0.706)
	sex	-0.005 (s.e.: 0.05; p: 0.924)	0.002 (s.e.: 0.05; p: 0.972)	-0.001 (s.e.: 0.051; p: 0.987)

outcome	beta estimates	base	smoking	symptomatic
	smoker_current	n.a.	0.165 (s.e.: 0.08; p: 0.04)	0.165 (s.e.: 0.081; p: 0.042)
	smoker_ex	n.a.	0.044 (s.e.: 0.058; p: 0.448)	0.042 (s.e.: 0.059; p: 0.473)
	symptomatic_yes	n.a.	n.a.	-0.024 (s.e.: 0.076; p: 0.75)
	symptomatic_maybe	n.a.	n.a.	-0.026 (s.e.: 0.065; p: 0.69)
	age*timeFU1	0 (s.e.: 0.001; p: 0.792)	0 (s.e.: 0.001; p: 0.897)	0 (s.e.: 0.001; p: 0.9)
	age*timeFU2	-0.001 (s.e.: 0.001; p: 0.433)	0 (s.e.: 0.001; p: 0.77)	0 (s.e.: 0.001; p: 0.763)
	sex*timeFU1	0.008 (s.e.: 0.042; p: 0.845)	0.004 (s.e.: 0.042; p: 0.926)	0.004 (s.e.: 0.042; p: 0.923)
	sex*timeFU2	-0.02 (s.e.: 0.042; p: 0.644)	-0.022 (s.e.: 0.042; p: 0.609)	-0.021 (s.e.: 0.042; p: 0.613)
	smoker_current*timeFU1	n.a.	-0.07 (s.e.: 0.076; p: 0.357)	-0.07 (s.e.: 0.076; p: 0.359)
	smoker_current*timeFU2	n.a.	-0.069 (s.e.: 0.073; p: 0.349)	-0.069 (s.e.: 0.073; p: 0.346)
	smoker_ex*timeFU1	n.a.	-0.001 (s.e.: 0.051; p: 0.981)	-0.001 (s.e.: 0.051; p: 0.985)
	smoker_ex*timeFU2	n.a.	-0.138 (s.e.: 0.051; p: 0.008)	-0.137 (s.e.: 0.051; p: 0.008)
	age*symptomatic_yes	n.a.	n.a.	-0.001 (s.e.: 0.004; p: 0.753)
	age*symptomatic_maybe	n.a.	n.a.	0.001 (s.e.: 0.004; p: 0.84)
log(S RBD IgG AI)	intercept	-1.22 (s.e.: 0.048; p<0.001)	-1.198 (s.e.: 0.054; p<0.001)	-1.15 (s.e.: 0.094; p<0.001)
	timeFU1	0.228 (s.e.: 0.029; p<0.001)	0.249 (s.e.: 0.033; p<0.001)	0.25 (s.e.: 0.033; p<0.001)
	timeFU2	0.36 (s.e.: 0.029; p<0.001)	0.357 (s.e.: 0.033; p<0.001)	0.357 (s.e.: 0.033; p<0.001)
	age	0.005 (s.e.: 0.002; p: 0.009)	0.005 (s.e.: 0.002; p: 0.008)	0.006 (s.e.: 0.004; p: 0.154)
	sex	-0.06 (s.e.: 0.066; p: 0.364)	-0.062 (s.e.: 0.066; p: 0.344)	-0.045 (s.e.: 0.064; p: 0.49)
	smoker_current	n.a.	-0.073 (s.e.: 0.093; p: 0.433)	-0.095 (s.e.: 0.093; p: 0.306)
	smoker_ex	n.a.	-0.055 (s.e.: 0.071; p: 0.435)	-0.075 (s.e.: 0.07; p: 0.287)
	symptomatic_yes	n.a.	n.a.	0.046 (s.e.: 0.103; p: 0.659)
	symptomatic_maybe	n.a.	n.a.	-0.115 (s.e.: 0.089; p: 0.195)
	age*timeFU1	-0.003 (s.e.: 0.001; p: 0.016)	-0.002 (s.e.: 0.001; p: 0.04)	-0.002 (s.e.: 0.001; p: 0.041)
	age*timeFU2	-0.003 (s.e.: 0.001; p: 0.002)	-0.004 (s.e.: 0.001; p: 0.002)	-0.004 (s.e.: 0.001; p: 0.003)
	sex*timeFU1	0.085 (s.e.: 0.039; p: 0.03)	0.083 (s.e.: 0.04; p: 0.036)	0.084 (s.e.: 0.04; p: 0.034)
	sex*timeFU2	0.036 (s.e.: 0.039; p: 0.363)	0.031 (s.e.: 0.04; p: 0.434)	0.031 (s.e.: 0.04; p: 0.435)
	smoker_current*timeFU1	n.a.	-0.052 (s.e.: 0.072; p: 0.467)	-0.052 (s.e.: 0.072; p: 0.469)
	smoker_current*timeFU2	n.a.	-0.009 (s.e.: 0.07; p: 0.894)	-0.009 (s.e.: 0.07; p: 0.9)
	smoker_ex*timeFU1	n.a.	-0.059 (s.e.: 0.049; p: 0.225)	-0.06 (s.e.: 0.049; p: 0.219)
	smoker_ex*timeFU2	n.a.	0.026 (s.e.: 0.049; p: 0.597)	0.026 (s.e.: 0.049; p: 0.602)
	age*symptomatic_yes	n.a.	n.a.	0.01 (s.e.: 0.006; p: 0.094)
	age*symptomatic_maybe	n.a.	n.a.	-0.006 (s.e.: 0.005; p: 0.244)
log(S ecto IgG)	intercept	-0.042 (s.e.: 0.046; p: 0.354)	-0.005 (s.e.: 0.05; p: 0.926)	-0.109 (s.e.: 0.089; p: 0.223)
	timeFU1	-0.161 (s.e.: 0.024; p<0.001)	-0.17 (s.e.: 0.027; p<0.001)	-0.169 (s.e.: 0.027; p<0.001)
	timeFU2	-0.299 (s.e.: 0.024; p<0.001)	-0.294 (s.e.: 0.028; p<0.001)	-0.294 (s.e.: 0.028; p<0.001)

outcome	beta estimates	base	smoking	symptomatic
	age	0.007 (s.e.: 0.002; p<0.001)	0.007 (s.e.: 0.002; p<0.001)	0.006 (s.e.: 0.004; p: 0.185)
	sex	0.011 (s.e.: 0.062; p: 0.861)	0.005 (s.e.: 0.062; p: 0.932)	0.02 (s.e.: 0.061; p: 0.749)
	smoker_current	n.a.	-0.243 (s.e.: 0.085; p: 0.004)	-0.241 (s.e.: 0.085; p: 0.005)
	smoker_ex	n.a.	-0.061 (s.e.: 0.066; p: 0.354)	-0.061 (s.e.: 0.066; p: 0.357)
	symptomatic_yes	n.a.	n.a.	0.204 (s.e.: 0.098; p: 0.039)
	symptomatic_maybe	n.a.	n.a.	0.07 (s.e.: 0.085; p: 0.413)
	age*timeFU1	0.001 (s.e.: 0.001; p: 0.552)	0.001 (s.e.: 0.001; p: 0.473)	0.001 (s.e.: 0.001; p: 0.482)
	age*timeFU2	-0.001 (s.e.: 0.001; p: 0.569)	-0.001 (s.e.: 0.001; p: 0.375)	-0.001 (s.e.: 0.001; p: 0.373)
	sex*timeFU1	0.047 (s.e.: 0.033; p: 0.163)	0.048 (s.e.: 0.033; p: 0.143)	0.048 (s.e.: 0.033; p: 0.144)
	sex*timeFU2	0.123 (s.e.: 0.033; p<0.001)	0.111 (s.e.: 0.033; p: 0.001)	0.111 (s.e.: 0.033; p: 0.001)
	smoker_current*timeFU1	n.a.	-0.009 (s.e.: 0.062; p: 0.885)	-0.009 (s.e.: 0.062; p: 0.882)
	smoker_current*timeFU2	n.a.	-0.085 (s.e.: 0.059; p: 0.151)	-0.085 (s.e.: 0.059; p: 0.152)
	smoker_ex*timeFU1	n.a.	0.031 (s.e.: 0.04; p: 0.444)	0.03 (s.e.: 0.04; p: 0.448)
	smoker_ex*timeFU2	n.a.	0.051 (s.e.: 0.041; p: 0.212)	0.05 (s.e.: 0.041; p: 0.217)
	age*symptomatic_yes	n.a.	n.a.	0.009 (s.e.: 0.006; p: 0.121)
	age*symptomatic_maybe	n.a.	n.a.	-0.002 (s.e.: 0.005; p: 0.738)
log(S ecto IgG AI)	intercept	-0.974 (s.e.: 0.048; p<0.001)	-0.945 (s.e.: 0.053; p<0.001)	-0.958 (s.e.: 0.094; p<0.001)
	timeFU1	0.193 (s.e.: 0.025; p<0.001)	0.189 (s.e.: 0.029; p<0.001)	0.189 (s.e.: 0.029; p<0.001)
	timeFU2	0.259 (s.e.: 0.025; p<0.001)	0.25 (s.e.: 0.029; p<0.001)	0.25 (s.e.: 0.029; p<0.001)
	age	0.004 (s.e.: 0.002; p: 0.023)	0.004 (s.e.: 0.002; p: 0.028)	0.004 (s.e.: 0.004; p: 0.315)
	sex	-0.072 (s.e.: 0.065; p: 0.269)	-0.079 (s.e.: 0.064; p: 0.219)	-0.065 (s.e.: 0.064; p: 0.311)
	smoker_current	n.a.	-0.237 (s.e.: 0.088; p: 0.007)	-0.249 (s.e.: 0.088; p: 0.005)
	smoker_ex	n.a.	-0.028 (s.e.: 0.069; p: 0.688)	-0.04 (s.e.: 0.069; p: 0.56)
	symptomatic_yes	n.a.	n.a.	0.094 (s.e.: 0.103; p: 0.364)
	symptomatic_maybe	n.a.	n.a.	-0.032 (s.e.: 0.089; p: 0.718)
	age*timeFU1	0 (s.e.: 0.001; p: 0.844)	0 (s.e.: 0.001; p: 0.952)	0 (s.e.: 0.001; p: 0.94)
	age*timeFU2	-0.001 (s.e.: 0.001; p: 0.213)	-0.001 (s.e.: 0.001; p: 0.176)	-0.001 (s.e.: 0.001; p: 0.174)
	sex*timeFU1	0.105 (s.e.: 0.034; p: 0.002)	0.109 (s.e.: 0.034; p: 0.002)	0.109 (s.e.: 0.034; p: 0.002)
	sex*timeFU2	0.08 (s.e.: 0.034; p: 0.019)	0.074 (s.e.: 0.034; p: 0.032)	0.074 (s.e.: 0.034; p: 0.033)
	smoker_current*timeFU1	n.a.	-0.033 (s.e.: 0.064; p: 0.608)	-0.033 (s.e.: 0.064; p: 0.607)
	smoker_current*timeFU2	n.a.	0.015 (s.e.: 0.061; p: 0.813)	0.015 (s.e.: 0.061; p: 0.81)
	smoker_ex*timeFU1	n.a.	0.018 (s.e.: 0.042; p: 0.664)	0.018 (s.e.: 0.042; p: 0.673)
	smoker_ex*timeFU2	n.a.	0.057 (s.e.: 0.042; p: 0.176)	0.057 (s.e.: 0.042; p: 0.179)
	age*symptomatic_yes	n.a.	n.a.	0.009 (s.e.: 0.006; p: 0.142)
	age*symptomatic_maybe	n.a.	n.a.	-0.004 (s.e.: 0.005; p: 0.447)

Supplementary Table 5: Association of symptomology and its interaction with age on eight antibody and avidity levels using linear mixed models. This table shows results from eight linear mixed models (LMMs), one for each of the following eight antibody and avidity levels as outcome: S RBD ELISA IgA, S RBD ELISA IgM, N NTD ELISA IgG, N CTD ELISA IgG, N NTD IgG AI, N CTD IgG AI, S ecto ELISA IgG, and S ecto ELISA IgG AI (ln-transformed to ensure approximate normal distribution). These results are based on an extension of the main model (see Table 3) by adding 2 symptomology variables (symptomatic_yes=symptomatic at the time of infection ascertained by positive PCR test, symptomatic_maybe=reported symptoms during the time period without ascertainment of specific time of infection by PCR test, with asymptomatic as reference meaning no symptoms during the time period until BL) and their age-interaction. This table shows covariate estimates, standard errors (s.e.) and P-values for the associations of the symptomology variables with outcomes (1st column) and their interaction with age (2nd column; age centered at 50 years). The analogous results for the other 5 levels are shown in Table 4.

	**Association with level coefficient (s.e.); P**	**Interaction with age coefficient (s.e.); P**
**S RBD IgA**		
age	0.007 (s.e.: 0.008; p: 0.371)	-
symptomatic_yes	0.371 (s.e.: 0.184; p: 0.044)	0.021 (s.e.: 0.01; p: 0.043)
symptomatic_maybe	0.277 (s.e.: 0.159; p: 0.082)	0.013 (s.e.: 0.009; p: 0.126)
**S RBD IgM**		
age	-0.01 (s.e.: 0.008; p: 0.223)	-
symptomatic_yes	0.126 (s.e.: 0.194; p: 0.514)	0.02 (s.e.: 0.011; p: 0.062)
symptomatic_maybe	0.262 (s.e.: 0.167; p: 0.117)	0.024 (s.e.: 0.009; p: 0.011)
**N NTD IgG**		
age	0.01 (s.e.: 0.008; p: 0.23)	-
symptomatic_yes	0.353 (s.e.: 0.187; p: 0.06)	0.019 (s.e.: 0.011; p: 0.077)
symptomatic_maybe	0.299 (s.e.: 0.162; p: 0.065)	0.002 (s.e.: 0.009; p: 0.792)
**N CTD IgG**		
age	0.003 (s.e.: 0.006; p: 0.623)	-
symptomatic_yes	0.424 (s.e.: 0.149; p: 0.005)	0.017 (s.e.: 0.008; p: 0.038)
symptomatic_maybe	0.244 (s.e.: 0.129; p: 0.059)	0.005 (s.e.: 0.007; p: 0.444)
**N NTD IgG AI**		
age	0.007 (s.e.: 0.004; p: 0.098)	-
symptomatic_yes	0.118 (s.e.: 0.097; p: 0.222)	0.004 (s.e.: 0.005; p: 0.51)
symptomatic_maybe	0.013 (s.e.: 0.083; p: 0.875)	-0.005 (s.e.: 0.005; p: 0.248)
**N CTD IgG AI**		
age	0.001 (s.e.: 0.003; p: 0.706)	-
symptomatic_yes	-0.024 (s.e.: 0.076; p: 0.75)	-0.001 (s.e.: 0.004; p: 0.753)
symptomatic_maybe	-0.026 (s.e.: 0.065; p: 0.69)	0.001 (s.e.: 0.004; p: 0.84)
**S ecto IgG**		
age	0.006 (s.e.: 0.004; p: 0.185)	-
symptomatic_yes	0.204 (s.e.: 0.098; p: 0.039)	0.009 (s.e.: 0.006; p: 0.121)
symptomatic_maybe	0.07 (s.e.: 0.085; p: 0.413)	-0.002 (s.e.: 0.005; p: 0.738)
**S ecto IgG AI**		
age	0.004 (s.e.: 0.004; p: 0.315)	-
symptomatic_yes	0.094 (s.e.: 0.103; p: 0.364)	0.009 (s.e.: 0.006; p: 0.142)
symptomatic_maybe	-0.032 (s.e.: 0.089; p: 0.718)	-0.004 (s.e.: 0.005; p: 0.447)
